# Compositionality in neural control: an interdisciplinary study of scribbling movements in primates

**DOI:** 10.3389/fncom.2013.00103

**Published:** 2013-09-12

**Authors:** Moshe Abeles, Markus Diesmann, Tamar Flash, Theo Geisel, Michael Herrmann, Mina Teicher

**Affiliations:** ^1^Gonda Brain Research Center, Bar Ilan UniversityRamat Gan, Israel; ^2^Department of Physiology, The Hebrew University of JerusalemJerusalem, Israel; ^3^Institute of Neuroscience and Medicine (INM 6), Research Center JülichJülich, Germany; ^4^Department of Applied Mathematics and Computer Science, Weizmann Institute of ScienceRehovot, Israel; ^5^MPI for Dynamics and Self-OrganizationGöttingen, Germany; ^6^IPAB, School of Informatics, University of EdinburghEdinburgh, UK; ^7^Emmy Noether Institute for Mathematics, Bar Ilan UniversityRamat Gan, Israel

**Keywords:** voluntary-movements, scribbling, compositionality, hand-motion-model, synfire chains, motion-primitives

## Abstract

This article discusses the compositional structure of hand movements by analyzing and modeling neural and behavioral data obtained from experiments where a monkey (*Macaca fascicularis*) performed scribbling movements induced by a search task. Using geometrically based approaches to movement segmentation, it is shown that the hand trajectories are composed of elementary segments that are primarily parabolic in shape. The segments could be categorized into a small number of classes on the basis of decreasing intra-class variance over the course of training. A separate classification of the neural data employing a hidden Markov model showed a coincidence of the neural states with the behavioral categories. An additional analysis of both types of data by a data mining method provided evidence that the neural activity patterns underlying the behavioral primitives were formed by sets of specific and precise spike patterns. A geometric description of the movement trajectories, together with precise neural timing data indicates a compositional variant of a realistic synfire chain model. This model reproduces the typical shapes and temporal properties of the trajectories; hence the structure and composition of the primitives may reflect meaningful behavior.

## 1. Introduction

The hallmark of human cognition is compositionality. We compose words out of phonemes, phrases out of words, sentences out of phrases. Primate dexterity exhibits the same property and may even form the evolutionary origin of language. Compositionality may be manifested either by stringing components along in time, such as in speech, or simultaneously, such as in understanding a visual scene. In motor behavior both forms are found abundantly. Complex drawing motions may be generated by concatenating simpler strokes. Picking up an object (*prehension*) requires simultaneous coordination of three time-dependent motions: arm reaching, hand orientation, and finger shaping.

In drawings, as in language, not all possible combinations are utilized. The rules—which stroke may be concatenated to which—constitute the syntax of action. Motor compositionality, again like in language, is manifested at multiple levels starting from the way we activate groups of muscles to produce a movement, and ending in the way we coordinate fingers, hand and arm when using a tool.

This article explores continuous two-dimensional scribbling motions as a compositional product of brain activity. It integrates mathematical modeling of scribbling, recordings of multiple single unit activities during scribbling, the syntax of concatenating scribbling primitives into a continuous drawing. It also develops neural network scribbling models that are consistent with the known psychophysics of hand motion control and the observable properties of single units in the motor cortex. Overall, it is shown how such a compositional approach may be applied to two-dimensional scribbling. It is based on the rationale that whereas an infinite plethora of drawings could be created, in practice there are only a limited set of shapes that are generated spontaneously. This is suggested by an intuitive analysis of the drawings of small children; here the principles are demonstrated for monkey drawings as well as for adult scribbling.

Our data indicate that in a well-trained monkey, scribbling is composed of a small number of elementary shapes concatenated into a continuous drawing. Human subjects under similar conditions tend to behave similarly. Such elementary components may be regarded as drawing primitives if it can be shown that they exhibit the following properties:

1. They are reproduced over and over again with a fair amount of consistency.
2. They are used as building blocks for more complex drawing.
3. They cannot be stopped in the middle.
4. They are related to specific activity states of the brain.
5. One can extract clear rules for their concatenation.

While other reported studies have attempted to identify primitives at the kinematic dynamic and muscular levels (see for example, Brandon et al., 2002; d'Avella et al., 2003; Mussa-Ivaldi and Solla, 2004; Polyakov et al., 2009a,b; Dominici et al., 2011; Overduin et al., 2012a,b) while still others have proposed computational models for inferring sub-movements and/or primitives and for modeling other aspects of motor compositionality (Thoroughman and Shadmehr, 2000; Giese et al., 2009; Degallier and Ijspeert, 2010; Hogan and Sternad, 2012; Ijspeert et al., 2013, and see the current special issue of frontiers in Neuroscience), relatively a few studies have searched for the neural correlates of primitives or sub-movements at the cortical or spinal cord levels (Hatsopoulos et al., 2007; Hart and Giszter, 2010; Overduin et al., 2012a,b). Here we combined behavioral, neurophysiological and theoretical studies and have investigated the issues of the inference of motion primitives, their neural representations and the syntactic principles subserving their combination into sequences. We have also suggested novel concepts concerning temporal and spatial aspects of brain representations of complex movements based on the notion of synfire chains. In particular, generating a complex drawing shape repeatedly calls for precise sequencing of muscular activities. We suggest a neural network structure which is compatible with the known anatomy of the cortex, and can generate the sequence of elementary shapes observed in the behavior. We show by way of simulations that such networks may indeed generate the observed behavior and provide indirect evidence for its existence.

Our presentation is built on two levels. In section 2 we provide a synopsis in which we describe the main facts that lead to the above description. Following this synopsis each part is described in greater details and more supporting evidence is provided.

## 2. Synopsis

Here we briefly describe our main results in the 7 sections. In section 2.1, we describe the monkey's scribbling data. In sections 2.2, 2.3 and 2.4 we describe various ways of analyzing the neural activities recorded while the monkeys were scribbling. In section 2.5 we describe a neural network that can produce scribbling similar to those of the monkey. Finally in sections 2.6 and 2.7 we analyse human scribbling and ways in which the syntax of concatenating simple strokelets into a complete drawing may be detected.

### 2.1. Monkey scribbling

#### 2.1.1. Materials and methods

Two monkeys (*Macaca fascicularis*) were trained to sit in a monkey chair and hold a low-friction, low-inertia manipulandum and continuously move it in the horizontal plane. The hand and manipulandum were under an opaque white screen so the monkey could not see them. A yellow dot was projected on the screen just above the position of the manipulandum's handle. The controlling computer was programmed to select a random (invisible) target in the working space. Once the monkey hit this target a beep was sounded, the monkey got a few drops of orange juice, and another target was selected at random. In this way the monkey was induced to move the manipulandum continuously. The *X*-*Y* position of the manipulandum was recorded at a rate of 100 per second. Target entry time, beep times and reward delivery were also recorded.

Once the monkey reached a stable behavior, it was anesthetized and prepared for recording of single-unit activities through metal microelectrodes. Spike shapes were detected by template matching either during the experiments or after it. Analysis of parallel spike trains in the cortex of behaving monkeys previously revealed a sequence of quasi-stable firing rate states (Radons et al., 1994; Abeles et al., 1995; Seidemann et al., 1996). This was also found to be true here.

#### 2.1.2. Results

**Figure 1:**
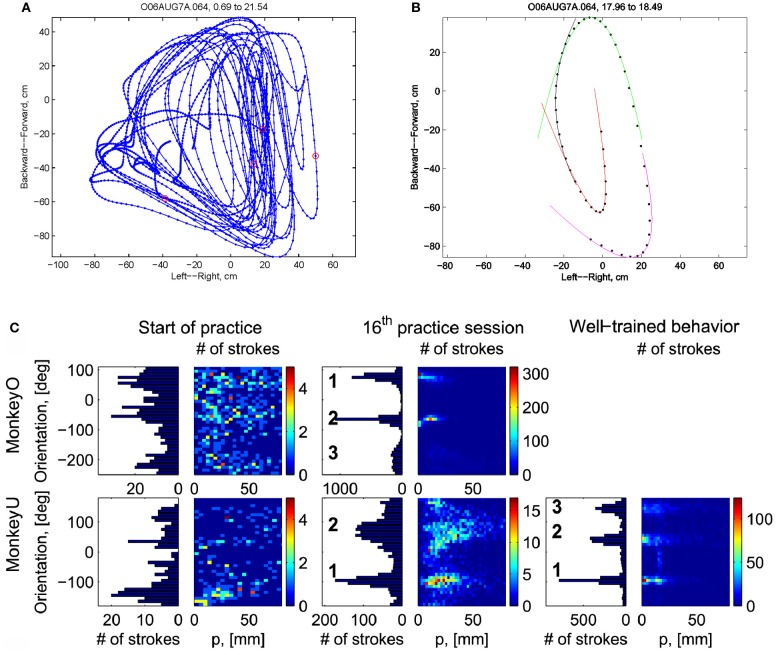
**Parabolic elements of scribbling. (A)** Sample drawing. Motion is sampled at 100/s (blue dots) points at which reward was given are marked by red circles. **(B)** Breaking a piece into 3 parabolas. Black dots—the measurements; red, green, magenta—three parabolas. **(C)** Changes over the course of training. For each parabolic segment two parameters were extracted: the orientation of the parabola and its focal distance. Those are plotted in the 2 dimensional (2-D) histogram. On the left is the histogram of the orientations (Y-marginal of the 2-D histogram). Each pair of histograms is for 1 training day. The left pair is for an early training session; the middle after a several months; and the right after half a year. Clearly, with training, three classes of parabolas emerged.

Initially the monkeys moved the manipulandum erratically but within several sessions the motion became rounded and more relaxed. Practice continued almost on a daily basis for a few weeks until the shape of the motion seemed stable. Figure 1A illustrates a short segment of a drawing. Red circles mark the points at which the monkey hit a target and got a reward.

Based on a previously suggested theory postulating the possible importance of equi-affine geometry in motion planning (Pollick and Sapiro, 1997; Handzel and Flash, 1999; Flash and Handzel, 2007), which was more recently extended to suggest a possible mixture of several geometries (Bennequin et al., 2009) it was postulated (Flash and Handzel, 2007; Polyakov et al., 2009a,b) that parabolas may subserve as drawings primitives. Indeed, we found that large segments of the drawings could be approximated by parabolas, where two parabolas are concatenated along their initial and final portions. Figure 1B illustrates such an approximation. The shape and orientation of a parabola may be specified by its focal distance (the radius of the circle at the tip) and the direction of its axis of symmetry. Figure 1C shows the distribution of these two parameters as the monkey's training progressed. Clearly, with time, the shapes “crystalized” into 2–3 parabolas.

Although the monkey often paused and occasionally drew some other shapes, the sequence of parabola #1 followed by parabola #2 followed by parabola #3 was by far the most prevalent.

If an entire parabola represents some elementary drawing component, there must be some expression in brain activity. This issue is examined in the next sections.

### 2.2. Hidden Markov model analysis

Drawing a large segment of a parabola requires recruiting neurons with different directional tuning in the correct sequence at the correct timing. If such a sequence is produced repeatedly it is likely that there will be brain activity that determines the drawing.

Producing a sequence of shapes repeatedly can be considered to be similar to the situation in speech when sequences of phonemes are repeatedly produced. The exact sound may vary from production to production, but the intention is the same. Speech analysis has benefitted from treating it as a Hidden Markov Model (Rabiner, 1989) where the intended phoneme is a hidden state, and the actual sound is an expression of this state. It is assumed that the hidden states behave like a first order Markov process. In such a process, time proceeds in small steps and at each step the state of the system either remains unchanged or flips to another state. The probabilities of transitions (or lack thereof) are specified by a matrix *P* that indicates the probability of changing from a state *i* to a state *j* for all pairs *(i, j)*. This process is hidden, but at each state the system emits some outputs. The probability of emitting each possible output *O* at each state *i* is given by an emission probability matrix *Q*. Once *P* and *Q* are known, for any observable set of emissions the most likely sequence of hidden states the system went through can be computed.

**Figure 2:**
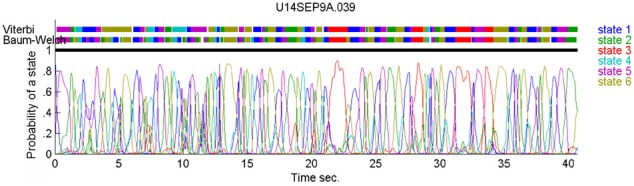
**Parsing data to HMM states.** Data for 41 s of scribbling is shown. Top bars give the sequence of hidden states as revealed by Viterbi training algorithm and Baum-Welch algorithm, respectively. The bottom graphs represent the probability of each possible state as a function of time (computed by the Baum–Welch algorithm). Most of the time there is only one dominant state and the transitions are steep. When no state had a probability above 0.5 the system was considered to be in an unknown state (left blank in the upper state bars).

In our case, the command or intention to draw a certain parabola can be considered to be a hidden state of the motor system. The brain activity during this state is the observed output. Applying Hidden Markov analysis to recorded brain activity may reveal hidden states that correspond to such intentions or commands Figure 2 illustrates a small time span showing the probability of being in any one of six states over this time span.

#### 2.2.1. Materials and methods

Once the monkey reached a stable behavior it was anesthetized and prepared for recording of single-unit activities through metal microelectrodes. Spike shapes were detected by template matching either during the experiments or after it. More technical details are described in section 3.

#### 2.2.2. Results

In the past, analysis of parallel spike trains in the cortex of behaving monkeys did reveal a sequence of quasi-stable firing rate states (Radons et al., 1994; Abeles et al., 1995; Seidemann et al., 1996). This was found to be true also in the present analysis. Figure 2 illustrates a small time span of analysis showing the probability of being at any one of six states over this time span.

It is often difficult to judge whether the analysis truly reveals some underlying states or is an inevitable result of the assumption that the observed firing is the outcome of a Markov process. In our data there is a unique opportunity to tackle this problem. In addition to the sequence of states we observed the behavior of the monkeys. If the HMM reveals states that are linked in time to the production of the parabolas we can infer that there are activity states which represent the intention (or maybe the plan or command) to draw the parabolas.

In this study we recorded the activity of only a few neurons out of tens of millions that are probably involved in planning and execution of arm motion. The recording area was in the arm region of M1 and the pre-motor cortices. Activities there are assumed to be related to the velocity vector of the hand end point. As such, they are expected to change dramatically over the span of each drawn parabola. A-priori the chances of finding stable firing configuration that span a whole parabola seem slim.

**Figure 3:**
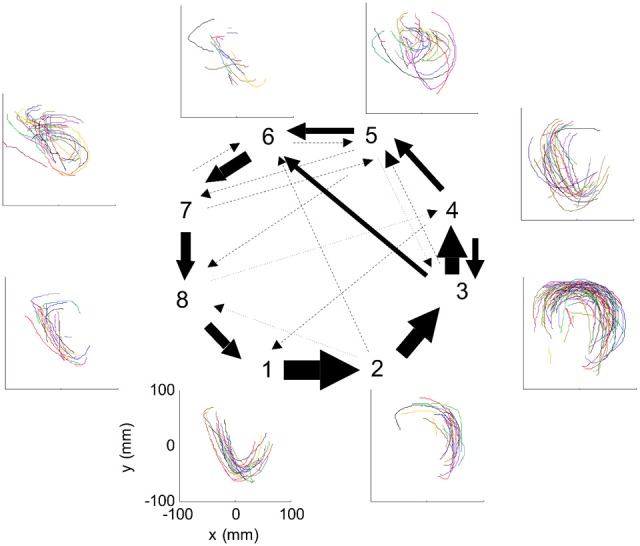
**HMM and drawings.** The transition probabilities between states are coded by the thickness of the arrows. The actual drawing shapes associated with each HMM state are plotted near the state number. The different colors have no meaning, they are meant to facilitate discrimination among the various repetitions of similar shapes. State 1 appears to be associated with one parabola, while states 2 and 3 with the two others. The other states are associated with other drawings, many of which had to do with pausing or restarting to move.

Nevertheless, in our data we found in four out of eight cases clear connection between the hidden Markov states and the parabolas. Section 4 describes this analysis in more details. Figure 3 illustrates the clearest case.

The findings that in some cases we found evidence of stable firing configurations that relate to the drawn parabolas support the notion that the parabolas represent a distinct entity in the monkey's scribbling. Is there any special significance for parabolas in hand motion? The next section addresses this issue from a mathematical point of view.

### 2.3. Equi affine geometrical analysis

Continuous two-dimensional (2D) drawings of human and monkeys obey a series of simple laws. One of them states that during continuous 2D drawings the drawing speed becomes slower as the curvature of the drawings become higher. The relation between angular speed and the curvature is described by a power law which may be written as:

$$A=KC^{\beta }$$

or by dividing by the radius (*R*):

$$V=KR^{\left(\beta \;-\;1\right)}$$

where *A* is the angular velocity, *C* is the curvature, *V* is the tangential velocity, *R* is the radius, *K* is the velocity-gain constant, and β is typically near 2/3. Note that *K* becomes larger when the drawing is larger (isochrony) and smaller when the level of accuracy is higher (Fit's law). There is something fundamental in the 2/3 power law as it holds not only for production of movement but also for the perception of motion speed. When a dot moves along a convoluted line, it is perceived as though it were moving at a constant speed when its movement follows the 2/3 power law (Viviani and Stucchi, 1992; Pollick and Sapiro, 1997; Levit-Binnun et al., 2006).

In differential geometry, the local properties of a curve can be described by means of the derivatives of the path with respect to some measure of distance (i.e., arc-length) and looking for invariant properties under some family of transformations. Of particular interest here are the equi-affine transformations in which a trajectory <*x*(*t*), *y*(*t*)> parameterized by time (or by arc-length) is transformed into a new trajectory <*u*(*t*), *w*(*t*)> by:

$$\begin{array}{l} u(t)=ax(t)\text{+}by(t)+c\\ w(t)=dx(t)\text{+}ey(t)+f \end{array}$$

with the condition: *ae* − *bd* = 1, i.e., the area within a closed loop is preserved by the transformation. When such trajectories follow the two-thirds power law in the time domain they have a constant equi-affine speed *K*.

**Figure 4:**
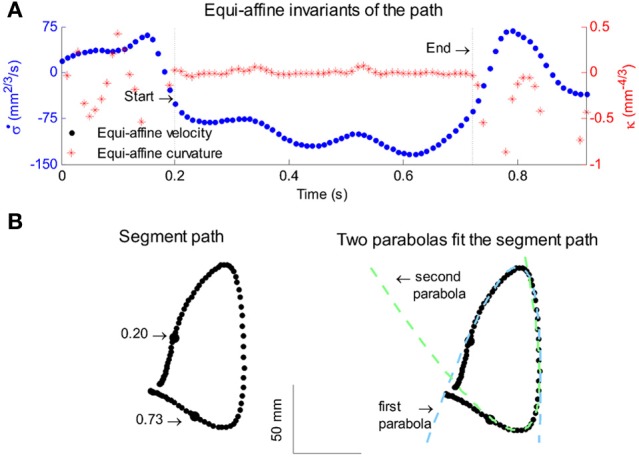
**Equi-affine differential geometry of monkey drawings. (A)** Equi-affine velocity (dots) and curvature (+sign) for a scribbling segment. **(B)** The actual drawing made by the monkey. Since parabolas are characterized by zero equi-affine curvature, the motion segments can be well-fitted by parabolas (dashed lines).

All parabolas have zero equi-affine curvature. Figure 4A illustrates the equi-affine properties of a short piece of a monkey's drawing. We observe half a second stretch [from time of 0.2 up to 0.73 at which the equi-affine curvature is close to 0 (red trace)]. This segment is composed of 2 parabolas as can be seen in Figure 4B. In fact it was such a finding that drew our attention to the fact that the drawings tended to be composed of sequences of parabolas. This issue is described in more detail in section 4.

Thus in equi-affine differential geometry parabolas play the role of straight lines in Cartesian geometry. The finding that the monkey drawing is composed to a large extent of parabolas, and that both motion production and the perception of the speed of a moving target obey the two-thirds power law which is equivalent to having a constant equi-affine speed, raises the possibility that at least some of the neuronal activity in the brain is coding the equi-affine parameters of the motion. This analysis is described in detail in section 4 and is summarized in the next section.

### 2.4. Tuning of single units in the motor cortex: partial correlations

It is well accepted that activity of neurons in the arm areas of M1 and premotor cortices code for the direction and velocity of motion (Georgopoulos et al., 1982, 1986, 1988; Moran and Schwartz, 1999), although there is strong evidence that they code also for lower level representation of movement [muscle group activity (Kakei et al., 1999)] and for higher level representations such as sequential order of movement sequences (Carpenter et al., 1999) or other more complex features of the motion (Paninski et al., 2004). Many studies of velocity tuning of cortical motor areas have been based on a simple task where the monkey needs to move from one starting location toward one of eight peripheral targets. In such a task the direction of the velocity vector, the direction of the initial acceleration vector and the position of the final target vary together. For this reason it may be difficult to distinguish which of the three components (position, velocity, acceleration) the unit is tuned to. In the current experiments, where the monkey is scribbling, as well as in experiments where the monkey had to trace convoluted trajectories, the coupling is less tight. However, in these experiments as well there are couplings between the parameters. These are even stronger if variable delays between the parameters are allowed because for quasi-periodic movements the velocity vector often looks like its derivative (acceleration) shifted in time. For this reason we developed the idea of partial correlation to find solutions for simultaneous parameters each of which are tuned to its own delay. For three parameters *x, y*, and *z* (here *x, y, z* stand for position, velocity, and acceleration) we describe the firing rate of a neuron at time *t* [λ*(t)*] as a linear combination of the effect of the three components with three delays:

(1) $$\lambda (t)=ax\text{​}\left(t+\tau_{x}\right)+by\text{​}\left(t+\tau_{y}\right)+cz\text{​}\left(t+\tau_{z}\right)+d$$

**Figure 5:**
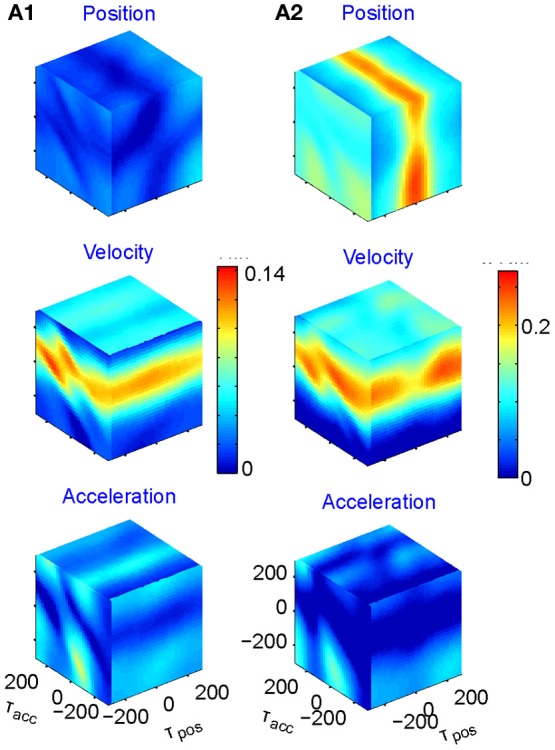
**Multi parameter tuning.** Data for single units in motor cortex of a monkey tracing a convoluted trajectory. Three possible parameters were studied: position, velocity, and acceleration. For each of them all possible delays within ±250 ms were tried. The color within the cube shows the contribution to the variance of firing rate for one parameter (e.g., velocity) given the other two (e.g., position and acceleration). **(A1)** Velocity tuning. This single unit showed only one plane of higher contribution to the total variance. At the velocity cube (middle) we see a horizontal plane (for τ_vel_) at time 70 ms (leading the velocity). **(A2)** Velocity and position tuning. For Position (given velocity and acceleration) we see a vertical plane at 0 delay and for the velocity (given position and acceleration) we see a horizontal plane leading the velocity by 90 ms. This single unit was coding two parameters at different delays.

We fit the best coefficients *a, b, c*, and *d* for every possible combination of the three delays in the range of ±250 ms, (taking into account that the rate cannot be negative) and test the fit (Stark et al., 2006, 2009). The results may then be displayed in a cube whose three dimensions are τ_*x*_, τ_*y*_, and τ_*z*_, with color reflecting the goodness of fit. However, as position, velocity, and acceleration may be highly correlated, it is better to build three such cubes for each neuron, one showing the regression on position when the contributions of velocity and acceleration are factored out; one for velocity when the contributions of position and acceleration are factored out; and one for acceleration when the other two are factored out. Figure 5 illustrates examples of such cubes.

Eighty percent (218 out of 272) of the units recorded were tuned to at least one of these parameters. The most prevalent was velocity (71% of the tuned units), 19% included acceleration, and 10% position. Fifty-six percent of the tuned units were tuned to only one of these parameters but only very few to all three. It is worth noting that when a unit was tuned to more than one parameter, the delays were generally different, as illustrated in Figure 5A2.

In a similar way velocity tuning may be related to the tangential velocity or the equi-affine velocity. These two parameters are very strongly correlated, so the distinction is less clear. The fit of the firing rate f(t) would look like:

(2) $$f(t)=a+b\dot{s}\left(t+\tau_{\dot{s}}\right)+c\dot{\sigma }\left(t+\tau_{\dot{\sigma }}\right)$$

Where *ṡ* and $\dot{\sigma }$ are the amplitudes of the Euclidian and equi-affine velocities, τ_*s*_ and τ_σ_ are the delays between firing and the Euclidian and equi-affine velocities.

**Figure 6:**
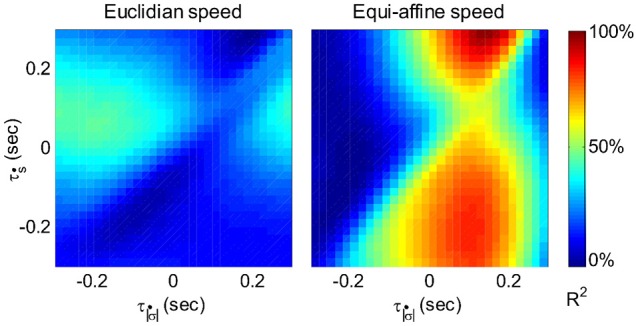
**Equi-affine tuning.** As Euclidian velocity and equi-affine velocity have the same direction only the amplitude of the velocity vectors was considered here. All possible delay combinations within ±250 ms were evaluated. The (thick) vertical line when equi-affine speed was the main regressor indicates that this unit is tuned to equi-affine speed. The thickness of the line may be attributed to the fact that both types of speeds are highly correlated; hence the contribution of one, after factoring out the effect of the other, is noisy.$\tau_{\left|\dot{\sigma }\right|}$ and τ_ṡ_ are the delays of the amplitudes of the equi-affine and Euclidian velocities.

Figure 6 illustrates a case with stronger tuning to equi-affine velocity.

In 6 out of 16 units for which this analysis was attempted, the tuning to Equi-affine velocity was stronger.

Even in M1 some single units may code not just for instantaneous position, velocity, or acceleration, but rather only for the serial order at which targets are presented, only to the direction of movement when the monkey moves its arm, or to both. Even when a single unit codes for both, the directions may be very different.

In our data, when the monkeys alternated between continuous curved motion (free scribbling or tracing) and center-out straight movements we found that 110 out of 304 units were directionally tuned during one of the tasks only, while only 72 during both. But even when a unit was directionally tuned in both tasks it did not necessarily have the same preferred direction. Thirty-eight of the seventy-two had preferred direction difference of more than 45°. Thus, representations in the motor cortex are far from uniform and heavily dependent on the context in which they are studied.

### 2.5. Simulation of neural networks for generating parabolas

#### 2.5.1. Introduction

We saw that the monkeys' scribbling tends to be characterized by concatenating parabolas and that parabolas are special shapes in terms of equi-affine differential geometry. We also know that many of the motor-cortex-units are tuned to the direction and velocity of the hand motion.

**Figure 7:**
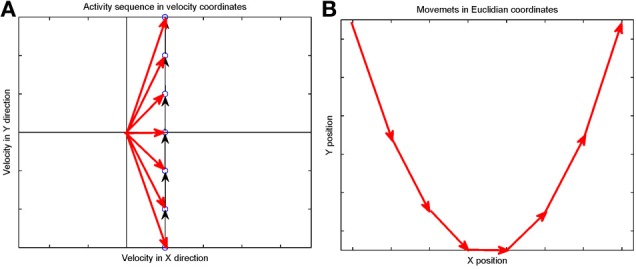
**Linear motion in velocity space. (A)** Progression through groups of neuron positioned on a linear line in velocity space (black arrows). The polar coordinates (red arrows) of each group describe the direction and amplitude of the motion in Euclidian space. Each circle represents a large group of neurons with the same velocity tuning. **(B)** The shape of trajectory that the progressing activity will generate. Since the position of the groups is linear in the velocity space the produced motion is a parabola. If the speed of activity- progression is constant, then the drawing will obey the 2/3 power low. If instead of seven groups of neurons [as in **(A)**] we would have many more in between, the parabolas [in **(B)**] would be much smoother.

Suppose that we plot these units in velocity coordinates (rather than in their topological position on the cortex). In such a plot the *(x, y)* position of the neuron is its velocity components in the *X* and *Y* directions. If we express its position in polar coordinates (φ, *v*) φ represents the direction of motion and *v* its speed. In these coordinates, motion with constant acceleration will be a motion on a straight line. By contrast, any straight line in these velocity coordinates represents motion along a parabola. This is illustrated in Figure 7.

Activity that moves at a constant acceleration along any such straight line in velocity space will represent motion along a parabolic trajectory while obeying the two-thirds power law.

If a neuronal network is behind the generation of a parabola it may be composed of groups of neurons that are activated one after the other, where each group of neurons has an appropriate location in velocity space. Each such group should be composed of neurons tuned for an appropriate direction and velocity such that when these groups are plotted in velocity coordinates they form a straight line. Activity should propagate with a constant delay from group to group. This description is consistent with the activity along a synfire chain. This notion was tested in simulations.

#### 2.5.2. Simulations

A synfire chain is essentially a feed-forward network composed of many pools of neurons. Each pool excites the following one by multiple diverging—converging excitatory connections. In such a network activity may propagate as a synchronous volley of spikes travelling through the pools or dissipate in time and quench (Abeles, 1982, 1991; Diesmann et al., 1999). Any individual neuron may take part in several pools of the same chain as well as in pools of other chains. Theoretical work suggests that if the pools are large enough (on the order of 100 neurons per pool or more) and the overall average activity is low enough (5 per s per neuron) each neuron may participate in many (10–100) pools without confusion (Bienenstock, 1995; Herrmann et al., 1995).

A simulation that mimics cortical tissue in which multiple synfire chains are embedded needs to include a situation where there is both excitation and inhibition with balance between them, so that the membrane potential of each neuron randomly fluctuates below threshold and only very occasionally hits the threshold and fires (Brunel, 2000). Each neuron should also receive multiple, possibly uncorrelated, inputs from other brain regions [on average the number of excitatory inputs arriving at any cortical patch is on the same order of magnitude as the number of neurons in the patch (Abeles, 1991; Braitenberg and Schuez, 1998)]. This implies simulating many tens of thousands of neurons with hundreds of millions of connections among them. Despite the theoretical work on the feasibility of embedding multiple synfire chains in a large network, in simulations the entire network tended to explode into global synchrony in a periodic manner and lost the identity of the individual synfire chains. To enable the embedding of several synfire chains in the network while maintaining spontaneous low levels of asynchronous background activity the homogeneity of neuromime properties had to be broken. In such a network each neuron can participate in several pools. In these conditions it is possible to excite individual synfire chains while assuring the propagation of a synchronous wave along each of the chains. More details are provided in section 7.

Consider a situation where each pool in a synfire chain codes for a different direction and velocity, such that when the pools' positions are plotted in velocity coordinates they lie along a straight line. A wave of activity progressing with a constant delay from pool to pool of the chain will thus produce a parabolic trajectory. A wave in a synfire chain may be elicited by synchronous excitation in one of its pools, or by enhanced, asynchronous, excitation to several of its initial pools. In the monkey, after long training, a particular sequence of parabolas tended to appear repeatedly. The parabolas became connected so that the end of one was tangent to the beginning of the other, conveying the impression of a smooth transition from one to the other.

In the above scenario the end of a synfire chain producing one parabola was at the same velocity-space location as the beginning of another synfire chain which produced another parabola. There may be several synfire chains starting (or passing through) that very same velocity space region, but with practice the end of one chain becomes connected in a stronger fashion to the beginning of another specific chain. These stronger connections between chains may be thought as the basis for the drawing syntax, where after a particular parabola *A* there is a higher probability of drawing another particular parabola *B*. If with training the monkey tends to produce sequence *A, B, C, A, …* the end of the network producing *A* becomes connected to the beginning of the network producing *B* whose end becomes connected to the beginning of the network producing *C* and so on.

**Figure 8:**
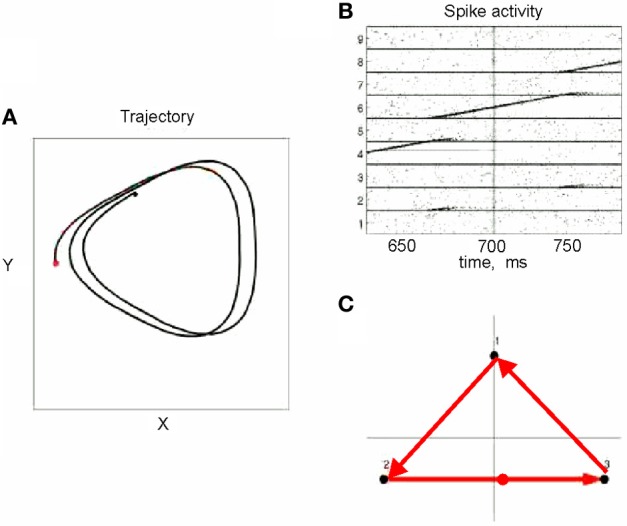
**Simulations of the production of 3 parabolas. (A)** The shape of the drawings generated by the simulation. **(B)** Raster plot of the activity in the network. Abscissa describes time, Ordinate provide the cell #. Activity of each neuron is described by dots along one line. The neurons along the ordinate are arranged according to their participation in the synfire chains. Synfire chains 4, 6, and 8 are the ones generating the drawing on the left. **(C)** The layout of the 3 synfire chains in velocity space. The synfire chain which is currently active is colored in thick red. At the moment activity is at the red dot producing the vertical motion near the maximal curvature of the parabola. Note that when one single synfire chain ends there is a competition among several others. E.g., at 750 synfire 6 ended and synfires 3, 7, and 8 show enhanced activity. After a short while synfire 8 wins.

This situation was simulated in a network of 50,000 neuromimes with 10 synfire chains three of which were connected as described above. Once activity was initiated in one of them it would circulate through the three producing a sequence of parabolas. Sample results of this simulation are illustrated in Figure 8.

If parabolic drawings are produced by activity waves in synfire chains, it is reasonable to inquire how these chains came about. Were they always there, explaining the tendency to draw parabolas? Or, perhaps whatever induced the monkey to draw the same parabola several times generated the connections between the pools of neurons with the appropriate velocity-tuning to become connected to each other by spike-timing-dependent-plasticity. Once such connections are established, even in a weak form, activity tends to follow the same pattern and strengthen the connections until they form a reliable synfire chain and produce a reliable segment of a parabola. We cannot answer this issue at this stage.

The simulations show the feasibility of generating the drawings by synfire chains which needs corroboration by experimental evidence. Is there any experimental evidence to support this idea? The next section deals with this issue.

#### 2.5.3. Experimental evidence

Direct observation of activity along a synfire chain requires the simultaneous recording of many neurons form the region in which the synfire chain is embedded. In simulations it was found that the activity of at least 200 neurons needs to be recorded simultaneously (Schrader et al., 2008). While recording techniques approach this limit, they typically record from a much larger volume than that in which a single synfire chain is expected to exist. The recording methods employed in the present study were unable to match the above figures. Thus, the most we can hope for is that by luck we record from two to three neurons that take part in the same chain.

If we record from two neurons that take part in a single chain, every time a wave sweeps through this chain we expect the two neurons to fire with some fixed interval in between. If the chain is associated with part of the monkey's drawing we expect to see this interval appearing repeatedly. In our data with approximately 10 neurons simultaneously measured and drawing quantized to approximately 22 different strokes we can construct 10 × 9 × 22 = 1980 different neuronal pairs for all the quantized drawings. If for each such pair we consider 50 possible intervals, there will be 50 × 1980 = 99,000 possible intervals, some of which will definitely contain a large number of repetitions even if there was no real preferred interval. To overcome this problem we adopted an idea proposed by Bienenstock and Geman (Geman et al., 2000; Amarasingham et al., 2003) as follows:

1. Define a statistics which you believe depends on the precision of intervals between spikes of different neurons. It should be one number that is extracted from the entire dataset.
2. Replace each spike time in the data by a random time within ±*W/*2 of the true time. If two spikes from the same neuron become closer than the refractory period, re-teeter their times till refractoriness is preserved.
3. Repeat all the calculations of the statistic on these teetered data.
4. Repeat steps 2 and 3 many (1000) times and build a histogram of the statistics of the teetered data.
5. Evaluate the probability of getting the statistics of the real data by finding what fraction of the histogram is equal to or larger than the real statistic.
6. Select increasingly smaller *W*. The value of the real statistic does not change, but the histogram of teetered statistics moves toward the real value as *W* becomes smaller. Find the smallest value of *W* for which the chance probability is smaller than the chosen significance.

This smallest *W* provides an estimate of the time precision of the data. Figure 23 illustrates the results of steps 1, 2, 3, and 4, for *W* = 10 ms. The precision obtained in this way is an upper bound on the spike-timing precision. Clearly, by defining a better statistics, a smaller W would be sufficient to obtain a significant difference between the real-data statistic and the teetered distribution.

This statistics, dubbed the “relations-score” was based on estimating the probability of getting *N* or more repetitions of each possible interval for each possible pair of neurons around each of the drawing strokelets. Minus the log of the product of the smallest 10 probabilities (*p*_*i*_) was defined as the relations-score:

$$\prime Relations-score\prime =-\sum_{i\;=\;1}^{10}log_{10}\left(p_{i}\right)$$

We took several measures to assure that large relations-scores would not be due to imperfect spike sorting, or merely to some strong correlations for one particular pair.

1. Only pairs recorded through different electrodes were used.
2. Each pair was allowed to contribute to only one of the smallest *p*_*i*_ used to compute the relations score.
3. As a control we selected the time sections for which the correlations are computed at random, rather than around specific shapes of the drawings.

**Figure 9:**
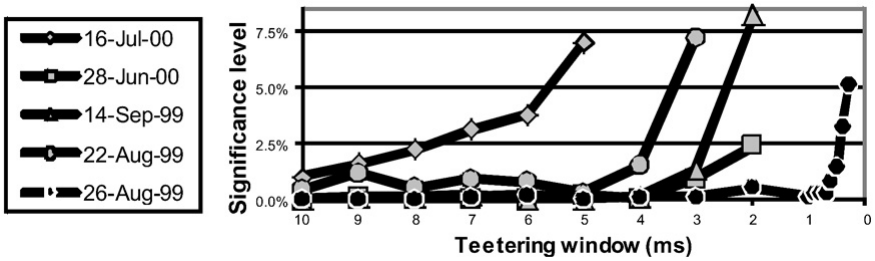
**Accuracy of spike intervals.** Significance level for different teetering windows is shown for each experimental day whose significance was at least 2.5% for 10-ms precision. Abscissa is the teetering window (from 10 to 0.3 ms); ordinate is the probability value of finding the relation-score by chance. Because we looked for significant values in drawing components based on both direction and velocity separately, all probabilities have to be multiplied by 2. That is, a significance of 2.5% in this figure stands for a significance of 5%, and so forth. As can be seen, the days have significant values for teetering at 0.5, 2, 2, 3, 4, 6, and 8 ms.

Eight experimental days in which the spike shape isolation was good and the firing rates stayed stable along the recording session were selected for analysis. This analysis was repeated for W of 10 ms and less, up to 0.3 ms. In five of the eight, accuracy was 8 ms or better. Figure 9 summarizes this analysis. If 5% is chosen as the significance level accuracy can reach 0.5 ms!

Accuracy did not reach even 10 ms on any day for randomly selected times during the recordings. Thus, the observed accuracy is clearly associated with the shape of the drawings. While these findings show that precise spiking intervals exist, and that they are associated with particular segments of the drawings, they do not prove that the drawings are produced or controlled by activity in synfire chains. However, we tried to disprove the hypothesis that the drawings were produced by synfire chains by looking for precise timing. Had we found none, the support for our hypothesis would be weakened. Nevertheless, we did find precise timing in five out of eight datasets at a precision of 0.5 ms. While these findings support our hypothesis, stronger support or disproof must wait until technology allows us to measure the spiking activity of many hundreds of neurons in a small volume in behaving subjects.

Elements of the drawings that repeat again and again can be produced by synfire chains. A movement primitive was defined as an entity that cannot be intentionally stopped before its completion (Polyakov et al., 2009a,b). A hint for such behavior was found in a well-trained monkey where the movement was usually decelerated after receiving a reward, but it stopped only after the completion of a sequence composed of several parabolic segments. A more direct test for the existence of “point of no return” in human scribbling is examined in the next section.

### 2.6. Point of no return: analysis of human scribbling

To study the existence of the “point of no return” we trained human subjects in the same setup as the monkeys. The subjects sat holding the manipulandum. They were told to keep moving it so as to hear as many sound beeps as possible. The working space was tiled with invisible hexagonal targets. Whenever the manipulandum handle passed through one of them a beep was sounded and another hexagonal target was selected at random. Some subjects tended to produce rounded movements while others moved essentially only to and fro (as the monkey initially did).

After a few sessions the task was changed. The subject was told to move, as before, but to stop immediately after hearing a beep and wait until told to start moving again.

**Figure 10:**
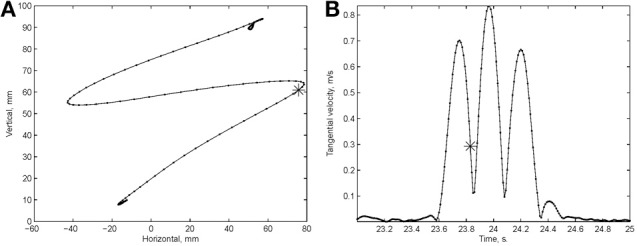
**Point of no return. (A)** The drawing. **(B)** The tangential velocity. At the star the stop sound was given. The subject continued to draw for half a second producing two additional peaks of speed.

Examination of the velocity of drawing indicated a succession of peaks with deep troughs in between. If stopping in the middle of the drawing caused no problems, we would expect the subject to stop at the next trough after the stop signal + processing delay. Figure 10 illustrates an example where the drawings continued for several up and down cycles after the stop signal, suggesting that the subject had to complete some pre-planned sequence of strokes before stopping. Such behavior was repeatedly observed in 6 out of 9 subjects. For more details see (Sosnik et al., 2007).

The existence of drawing elements that repeats over and over again in scribbling, and the finding that some cannot be stopped in the middle support the notion that these represent some sort of drawing primitives. The idea that each such primitive is generated by an activity wave propagating through a synfire chain is attractive in the sense that it explains the sequencing in time of the series of small movements (strokelets) that compose the primitive and explains why it is very difficult to stop such elements of motion in the middle. However, the available data do not either prove this idea or disprove the possibility that some other type of neural network is responsible for producing such elements. The scribbling was often composed of several distinct repeating elements and tended to show regularities in terms of the order of recruitment of these elements. These regularities may be treated as the “syntax” of scribbling. The next section discusses how this syntax can be detected.

### 2.7. The syntax of scribbling

**Figure 11:**
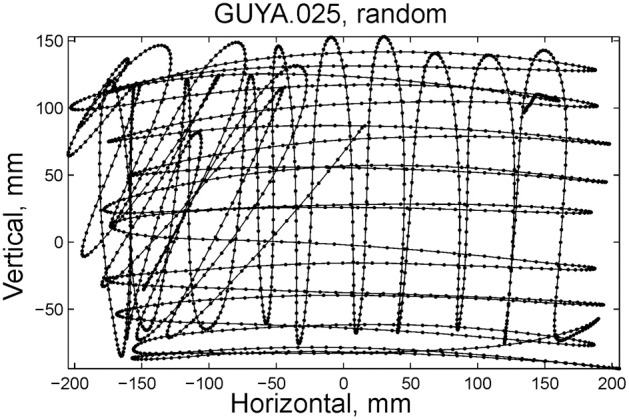
**Sample of scribbling.** Scribbling was sampled at 100/s. The subject scribbled until told to stop. Several such segments were concatenated for the analysis.

Some of the human subjects carrying out the scribbling tasks tended to move to and fro in a regular manner. Figure 11 illustrates such a scribbling session. To the human eye and brain this strategy is quite clear. The subject is scanning the work space horizontally and once finished, scans it vertically and then horizontally again etc. … Can this structure be revealed automatically?

We start the analysis by breaking the scribbling into smaller segments (strokelets). The velocity of scribbling shows multiple peaks separated by deep troughs. It is reasonable to assume that each section between two successive velocity minima should be considered as one segment. Nevertheless, in the monkeys' scribbling the different parabolas were concatenated at points of maximal velocity. Lacquaniti et al. (1983), showed that when parsing tracing motion according to the 2/3 power law, one obtains segments with close- to- constant velocity gain coefficients (*K*) with abrupt shifts in *K* at the points of maximal velocity. For these reasons we decided to parse the scribbling between adjacent extrema in the velocity.

**Figure 12:**
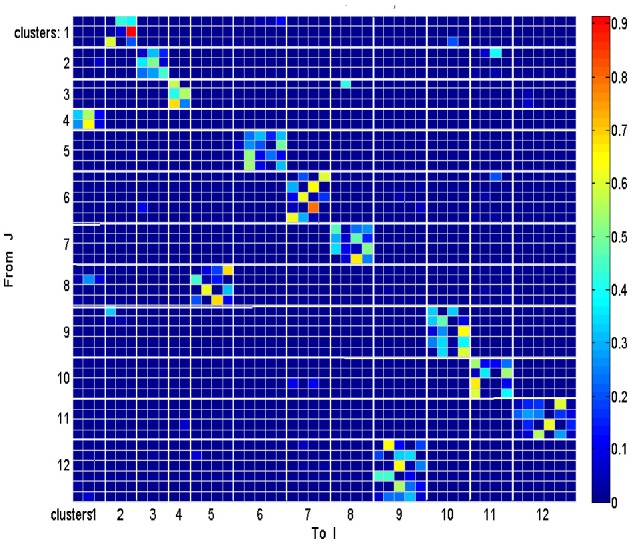
**Conditional transition matrix**.

Each such strokelet was then described by the direction of motion at 10 points along its trajectory, and whether it was accelerating or decelerating during the strokelet. The strokelets were clustered into 47 clusters, 23 for accelerating strokelets and 24 for decelerating. We then used the information bottleneck analysis (Tishby et al., 1999; Slonim and Weiss, 2000) to cluster these 47 groups so as to preserve the maximal mutual information between each strokelet and its subsequent one. The results are depicted in the matrix in Figure 12. Cluster 1 (top left in the matrix) is composed of 3 groups of strokelets. They are followed by strokelets in cluster 2 which are followed by strokelets in cluster 3 which are followed by strokelets in cluster 4 which are followed by strokelets from cluster 1. Thus, with a few exceptions cycles of clusters 1→ 2→ 3→ 4→ 1→ … This could be compared to spoken language. Each of the 47 groups is like a phone. A cluster of phones with similar transition properties is like a phoneme, and the sequence of frequently connected phonemes (e.g., 1, 2, 3, 4) is like a word. In this analogy, there are three words: A composed of clusters 1, 2, 3, and 4; B composed of clusters 5, 6, 7, and 8; C composed of clusters 9, 10, 11, and 12. The sentences in this analogy are: “α” composed of the sequences A, A, A,…; or “β” composed of B, B, B,…; or “γ” composed of C, C, C,… The transition between sentences is not random. Sentence β is followed only by sentence α. This transition occurs only when one of the group members (“allophones”) of cluster 8 is followed by one of the group members of cluster 4 in sentence α. Similarly, sentence γ is followed by sentence α only when one of the group members of cluster 9 is followed by one of the group members of cluster 2 in sentence α. Sentence α, on the other hand may be followed by either sentence β or γ. Here too the transitions are through one specific “allophone” from cluster 3 in α into cluster 8 in β; or from cluster 2 in α into cluster 11 in γ. Thus, the structure of a paragraph in this analogy is: β, α, γ, α, β,…

Sentence β is composed of left-right-left … strokes, sentence γ is composed of up-down strokes and sentence α of diagonal strokes. While this is a very simple example it shows that there is some internal “syntax” for the scribbling and illustrates how this syntax can be revealed without human interpretation.

The language of scibling in monkeys and men is very limited. The above method would probably be insufficient to reveal the syntax of handwriting. Yet this analysis shows that scribbling does have its internally driven syntax.

More details on this analysis can be found in section 8.

### 2.8. Summary

This overview described how scribbling is generated, the brain activity correlates of scribbling, what neural networks can generate the scribbling and how the rules by which simple elements of scribbling are concatenated when a longer drawing is generated. Although each of these topics deserves fuller exploration, these findings are sufficient to support our hypothesis that synfire-chain-like structures are likely to underlay the neural data. It can conversely reproduce the behavioral data.

## 3. Data acquisition

### 3.1. Materials and methods

Monkey subjects (*Macaca fascicularis*) were trained to hold a low friction and low inertia manipulandum and carry movements in the horizontal plan. The subjects could not see their hand or manipulandum. An opaque white screen was positioned just above the manipulandum and a cursor (yellow circle) was projected on a point just above the manipulandum's handle. When necessary, additional targets were projected too on the same screen. A juice spout was in touch with the subject lips. The desired behavior was reinforced by releasing a few drops of orange juice whenever the subject successfully completed a trial. No negative reinforcements (punishments) were employed.

Three types of experiments were performed: Free scribbling, tracing and a center-out task. In the free scribbling the subject was motivated to continuously move the manipulandum in the following way. A target zone (invisible to the subject) was selected at random. When the target was hit, a short beep was heard, the subject was rewarded, and the target jumped to another random location.

In the tracing task, a trial was started by projecting a green target on the opaque screen in front of the subject. As soon as the subject brought the cursor into this green target, a convoluted trajectory was displayed in gray. The initial target disappeared and an elongated target made out of eight partially overlapping green circles was displayed on the gray trajectory just in front of the initial target. Once the subject placed the cursor into the first circle it disappeared and another circle was added in front of the elongated target. In this way it appeared as if the subject was chasing an elongated worm that progressed along the convoluted trajectory. When the subject reached the end of the trajectory it was rewarded by a few drops of orange juice. On each day a set of different 40 trajectories was selected out of a repertoire of 100 trajectories. The trajectories were generated by spline interpolation between 10 randomly selected points.

### 3.2. Surgery and monkey handling

Following training, a localizing MRI scan was performed and a chamber (22 × 22 mm) was implanted in aseptic conditions [halothane anesthesia, induced by ketamine and medetomidine hydrochloride (Domitor)] over the left hemisphere. The dimensions of the chamber were selected in order to allow access to both motor and premotor areas. Analgesia [pentazocine (Talwin), carprofen (Rymadil)] and antibiotics (ceftriaxone) were administered peri-operatively. The dura mater was left intact. Location of sulci was confirmed visually during surgery and, following chamber implantation, by another MRI scan.

All the procedures were supervised by the institution veterinary, approved beforehand by the institutional ethics committee and conformed the laws in Israel, and the NIH Guide for the Care and Use of Laboratory Animals (1996).

### 3.3. Neural and behavioral recordings

During each recording session up to eight glass-coated tungsten micro-electrodes (impedance 0.2–2 M at 1 kHz) were inserted through the dura. Electrodes were arranged in a circular guide tubes (MT, Alpha-Omega Engineering, Nazareth, Israel), such that inter-electrode spacing within a circle was ~ 300 μm. Each electrode was moved independently (EPS 1.31, Alpha-Omega Eng.). The electrodes were inserted either into the primary or the premotor areas. The signal from each electrode was amplified (10 K), band-pass filtered (1–6000 Hz), and sampled at 25 kHz (Alpha-Map 5.4, Alpha-Omega Eng.). Eye movements were recorded using an infra-red beam system (Oculometer, Dr. Bouis, Karlsruhe, Germany) tracking movements of the right eye. The 2D signal from this system, as well as the position of the robotic arms, was sampled at 100 Hz. Behavioral events (LEDs, switches, lights, and so on) were sampled at 6 kHz. The workspace and monkey's movements were monitored using three infra-red CCD video cameras synchronized to the task and recorded on computer disk.

### 3.4. Neural data preprocessing

An offline procedure was applied to identify spike waveforms in the 25 kHz digitized traces (Bar-Hillel et al., 2006). Spikes were subjected to manual offline spike-sorting (Abeles and Goldstein, 1977) (Alpha-Sort 4.0, Alpha-Omega Ind.), and the clusters defined examined for unit isolation (ISI histograms, individual spike shapes) and unit separation (Ben-Shaul et al., 2003). Long-term (trial-to-trial) stationarity of the responses of each unit was determined by an algorithm based on a time-varying Poisson counting process and validated by visual inspection of raster plots.

Further details on the methods of analysis of the behavior and spike trains are described in the appropriate places of the following sections.

## 4. Geometric approach to movement analysis

### 4.1. Piece-wise parabolic patterns emerge in spontaneous over-trained primate drawings

Since the pioneering work of Bernstein (1967), there has been a general consensus that we have mental templates of motions which we try to follow when executing motor tasks. For example, reaching movements consist of fairly straight lines with bell shaped velocity profiles. Several optimization criteria have been suggested as the basis for the selection of these motion primitives (Flash and Hogan, 1985). The shape of the velocity profiles is invariant under changes in speed (Atkeson and Hollerbach, 1985) unless there are accuracy demands, in which case the movements obey Fitts' law (Fitts, 1954).

Simple curved motions which contain several velocity peaks may be observed during obstacle-avoidance movements or when the target jumps in the middle of the motion. Such motions seem to be composed of the summation of straight or slightly curved motions, which are partially overlapped in time (Morasso and Mussa-Ivaldi, 1980). Similarly, the analysis of movements generated by stroke patients or during load adaptation tasks has shown that template for the speed of hand trajectory might be composed of a single or a few velocity primitives (Krebs et al., 1999).

Continuous two-dimensional drawing motions tend to follow a power law (Lacquaniti et al., 1983) *A* = *KC*^β^, where *A* is the angular velocity, *C* is the curvature, the power β is often near 2/3, and *K* is a gain factor which has been shown to be piecewise constant. The value of K depends on the linear extent of the segment, in a way that conforms to the isochrony principle (Viviani and Schneider, 1991). Thus, in spite of the apparent continuity of drawing movements, they may be, in fact, intrinsically discontinuous and constructed of individual segments. We term such intrinsic components as “primitives” of motion. While there is experimental evidence supporting the idea that complex motion is composed of primitives (Flash and Hochner, 2005; Hart and Giszter, 2010), not everybody agrees on that (Tresch and Jarc, 2009).

To examine the nature of movement elements from which monkey scribbling movements are constructed, three data sets recorded from two monkeys were analyzed and included two data sets recorded during 16 or 17 sessions at the beginning of the practice period and one data set recorded from one of these monkeys during 17 sessions conducted following a full year of practice. Kinematic analysis of the scribbling movements of the highly trained monkey showed that these movements can be well-approximated by parabolic segments. The movements were first segmented into periods of rest and active drawing and the drawing movements were then kinematically analyzed and various geometric, temporal and kinematic variables were calculated. These variables included hand velocity and acceleration, Euclidean arc-length and curvature and equi-affine arc-length curvatures.

To empirically examine whether the monkey movements can indeed be shown to be composed of a sequence of parabolas, the movement records were segmented into separate strokes, each lying between local minima of Euclidean curvature, and containing a single maximum of Euclidean curvature. These strokes were then fitted with parabolic segments whose canonical representation is $y=\frac{x^{2}}{2p}$. The parameter *p* is the focal parameter of the parabola, its value being equal to the radius of curvature at the point of maximum curvature. For more details see Polyakov et al. (2009a,b).

The error in fitting a stroke with a parabolic model was estimated using the parameter D evaluating the proportion of the data variance unexplained by the parabolic model, namely:

$$D=1-R^{2}=\frac{\sum^{\text{​}}{\left(y_{i}-\frac{x_{i}^{2}}{2p}\right)}^{2}}{\sum^{\text{​}}{\left(x_{i}-\text{mean}\left(x\right)\right)}^{2}+{\left(y_{i}-\text{mean}\left(y\right)\right)}^{2}}$$

### 4.2. Parabolic patterns during drawing: evidence from monkey scribbling

**Figure 13:**
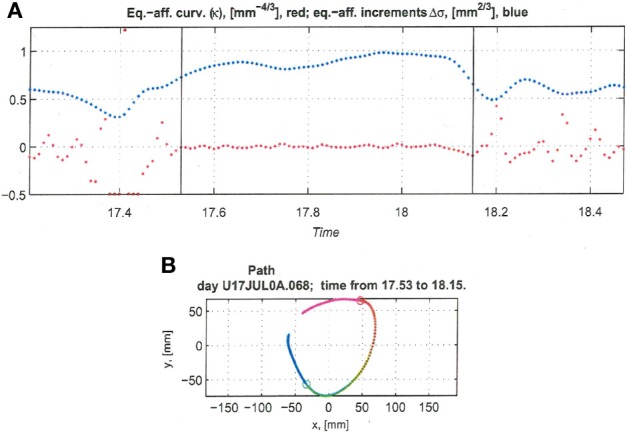
**Equi-affine analysis of drawing and parabolic fit.** A short stretch of the monkey's drawing is shown. **(A)** Equi-affine analysis shows that between time 17.53 and 18.15 the equi-affine curvature (red) was almost 0. **(B)** Fit to parabolas was close to perfect. The section between 17.53 and 18.15 is between the green and red circles. It is fitted by two parabolas painted red and green.

**Figure 14:**
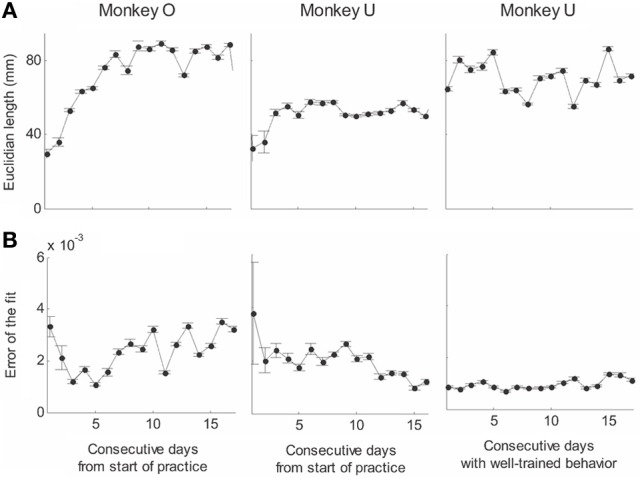
**Degree of fit and length of the parabolic strokes fitted to movement segments. (A)** Euclidean lengths of the fitted parabolic strokes. In each plot, median values (over sessions) and 95% confidence intervals are shown. **(B)** Values of the D parameter estimating the error in fitting parabolas to the extracted segments.

Recorded trajectory segments were fitted with parabolic strokes (see Figure 13). The length of the movement segments that were well-approximated by parabolas was found to be longer for data derived from well-trained behavior vs. those derived from movements performed during the beginning of the practice period (see Figure 14A). Figure 14B shows the values of the D parameter estimating the error in fitting parabolas to the extracted segments. As is clear from this figure such error became smaller as a function of the amount of practice the monkeys have had. To assess the degree to which scribbling movements are well-approximated by parabolic-like strokes, the values of the equi-affine curvature along these strokes were derived (see also Figure 4A) and their modifications with practice were examined.

**Figure 15:**
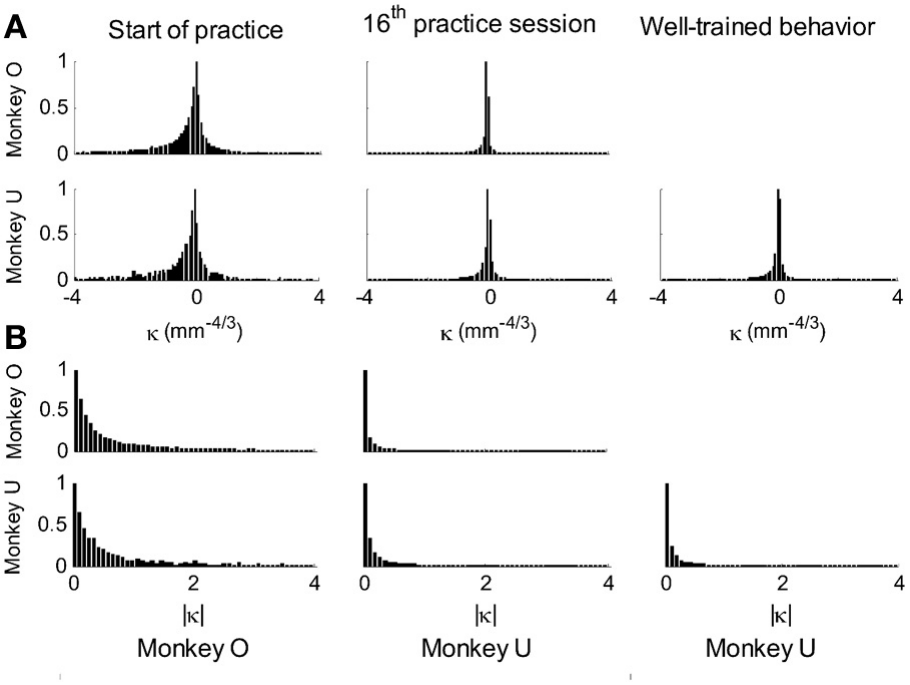
**Equi-affine curvature and its modifications with practice. (A)** Distributions of equi-affine curvature (measured in mm^−4/3^) for the first recording session, a session conducted following 16 practice days, and another session of well-trained behavior. **(B)** Distributions of the magnitude of the equi-affine curvature (same data).

This analysis showed that the distributions of the equi-affine curvature k of the fitted strokes peaked at zero (histograms in Figure 15A) with both negative and positive values. Moreover it was found that during the first five to six practice sessions, the absolute values of the equi-affine curvature |*k*| consistently decreased, converging toward nearly zero equi-affine curvature (Figure 15B). Hence, with practice, the extracted movement segments indeed tended to become more parabola-like. We also assessed what other geometric forms besides parabolic strokes may possibly provide a good fit to the extracted scribbling segments. These other geometric forms included ellipses and polynomials of third, fourth, and fifth order. Several considerations suggest that parabolic rather than elliptic or polynomial segments provide a better model for drawing movements [for further details see Polyakov et al. (2009b)]. To quantify the trade-off between goodness-of-fit and model simplicity (number of parameters of the fitted curve) the Schwarz information criterion (SIC) (Schwarz, 1978) was used (Polyakov et al., 2009b). This analysis showed that the parabolic model yielded the highest SIC score indicating that the parabolic model is optimal in the sense of goodness-of fit vs. simplicity trade-off. Hence, taken together these results suggested that parabolas might be considered as more attractive candidates for serving as plausible movement primitives.

### 4.3. Clustering of the extracted parabolic segments

**Figure 16:**
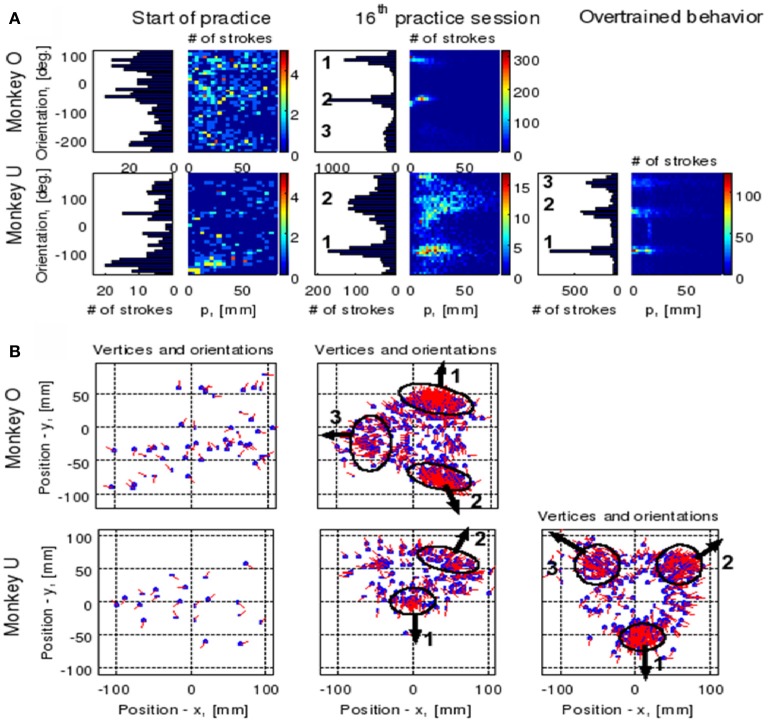
**Emerging parabolic clusters and dimensionality reduction. (A)** Typical histograms for the fitted parabolic segments. In the one-dimensional histogram (left), the segments are counted according to their orientation. In the color histogram (right), they are counted in distinct bins according to the orientation and focal parameter of the parabola. **(B)** Location of the vertex and orientation of the parabola for every 10th parabolic segment for the recording sessions in **(A)**. Locations of the vertices of the similarly oriented parabolas are also clustered. The clusters are marked by ellipses and the mean orientations of the parabolas within each cluster are depicted by arrows.

The focal parameter and the orientation define a unique parabola up to translation (see Figure 16). The parabolic segments that were fitted to the recorded movement segments were then clustered into different clusters according to their spatial orientation. In comparison to the lack of distinct clusters in the histograms obtained for the parabolic segments derived from the movements recorded during the beginning of the practice period the parabolic segments extracted from the well-practiced movements clearly showed convergence toward a few well-separated clusters (see Figure 16). Hence the well-practiced movements could be fitted by three parabolic segments (see Figure 16B). Further examination of the locations of the vertices of similarly oriented extracted parabolic segments also showed that after a period of practice, these locations could be separated into three distinct locations.

### 4.4. Neural coding by means of equi-affine variables

While the above analysis has indeed supported the hypothesis that parabolas might be considered as likely candidates for serving as movement primitives, further analysis was carried out to directly examine whether equi-affine speed is indeed represented in single unit motor cortical activities recorded during well-trained scribbling. This analysis was based on the method suggested by (Stark and Abeles, 2007). Here we describe the procedure used to compare the representation strengths of Euclidean vs. equi-affine speeds [for details of the method see Stark and Abeles (2007) and Polyakov et al. (2009b)].

Given that the equi-affine and Euclidean speeds were found to be highly correlated for the recorded scribbling movements the neural activities related to one of these two variables is expected to be trivially related to the other variable at a similar time lag. Hence, to overcome this problem, the relation between single-unit firing rates vs. Euclidean and equi-affine speeds were simultaneously analyzed at multiple time lags using the following multiple linear regression model as described in Equation 2 of section 2.4:

(3) $$f(t)=a+b\dot{s}\left(t+\tau_{\dot{s}}\right)+c\dot{\sigma }\left(t+\tau_{\dot{\sigma }}\right)$$

where *f*(*t*) is the unit's firing rate at time *t*, τ_*ṡ*_ and $\tau_{\dot{\sigma }}$ are the time lags for the Euclidean and equi-affine speeds, respectively, and *a, b*, and *c* are regression coefficients. Positive time lags correspond to the neural activity preceding the movement. Note that Euclidean and equi-affine velocity vectors have the same direction. Therefore, we ignore the direction of the velocity vectors and relate only to the amplitude of these vectors. The influence of the Euclidean and equi-affine speeds was estimated using the measure of contribution defined in (Stark et al., 2006).

Following Stark et al. (2006) [for further details see Polyakov et al. (2009b)] a stripe (horizontal or vertical set of values) in a contribution matrix, all corresponding to the same time-lag of one of the two speed parameters was deemed dominant if at least half of the constituent values were above *max*(*R*^2^)/2, where the maximum is taken over all *R*^2^ values at all time-lag combinations of the two parameters. Using this method, the activity of 87 well-isolated units recorded during the scribbling task was analyzed. The activity of 72 units (83%) was related to the monkey's hand position, velocity, acceleration, or some combination of these kinematic variables.

**Figure 17:**
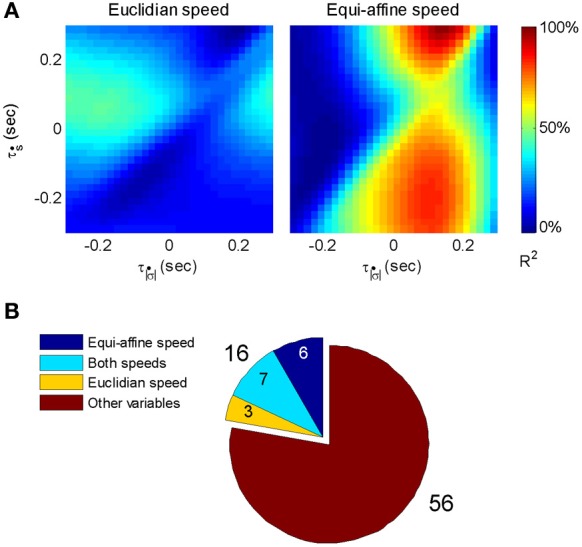
**Motor cortical activity related to equi-affine speed. (A)** Contribution matrices, used to compare the representation strength of Euclidean (left) and equi-affine (right) speeds in the activity of one motor cortical unit. Contributions are shown as fractions of the total (0.12 in this case) of a linear model including both speeds (see Equation 3). The vertical stripe in the right matrix indicates that the equi-affine speed is more strongly represented. The stripe appears at a time-lag of 0.12 s, where neural activity precedes the movement. This part was reproduced in Figure 6. **(B)** Number of movement-related units whose activities receive dominant contribution from the equi-affine and/or Euclidean speeds.

The contribution matrices for the combined speed model for one of these units are shown in Figure 17A. For the unit depicted there, the firing rate is movement-related because contribution of equi-affine speed to the firing rate variance is dominant as the right matrix contains a vertical dominant region around the time lag of 0.12 s, indicating that neural activity precedes movement (permutation test, *P* < 0.05). In contrast, the contribution matrix for the Euclidean speed (left) does not contain a dominant stripe. Further analysis showed that the combined Euclidean/equi-affine speed model (see above) provided a good fit for the firing rates of 16/72 (22%) of the movement-related units (permutation test, *P* < 0.05; Figure 17B). The activity of seven of these units (44%) was related to both Euclidean and equi-affine speeds. However, equi-affine speed was dominant in the activity of six units (38%) whereas Euclidean speed was dominant for only three units.

## 5. Analysis of neural states in parallel spike trains

In this section we wish to treat the parallel recorded spike train as a time-varying vector of activity. We do this by considering the recorded activity as the output of a Markov chain.

In a first order Markov chain a system may be in one of *N* states while time progresses in discrete steps. For each state there is a vector of probabilities that the system would flip to any other state or remain where it is now. The state of the system may not be known explicitly, but is observable by some output that is related to the state in a probabilistic manner. The observed information can be disentangled by an estimation of the most likely state by a Hidden Markov Model (HMM). HMMs were used for the analysis of spike trains in the past (Radons et al., 1994; Abeles et al., 1995; Gat et al., 1997). In our analysis, we assume that the little piece of cortex in which our electrodes were placed behaves like a Markov chain, and the activity of several (M) recorded neurons is the observable output of the system. Thus, we assume that the piece of cortex may be in one of *N* states, we first find an optimal *N* × *N* matrix of the probability for transitions among states (*P*) and an optimal set of M firing rates at each state (An *N* × *M* matrix of firing rates Λ). As firing rates are typically low, time steps had to be fairly big (50 ms). We will assume that the probability of observing *n* spikes of a certain single unit is: *p*(*n|x*) = *e*^−*x*^ *x*^*n*^/*n*!, where x is the expected number of spikes given by x = λΔ*t*, where λ is the firing rate and Δ*t* is the duration of the step. We further assume that the different single units independently fire.

With these assumptions and knowing P and Λ, we may estimate the likelihood of observing the measured spike trains throughout the recorded period for any possible series of the states of the Markov chain. We start with a guess of P and Λ and improve them by expectation maximization procedure. Here we used both the Viterbi training algorithm and the Baum-Welch algorithm, and once optimal P and Λ are found we can also specify the state-sequence that provides the best likelihood of observing the recorded spike train. Typically, for any given spike train, within a window of 50 ms, no spikes at all were observed in many cases, occasionally there was one spike, and rarely two or three. This situation produces a very uneven terrain of likelihood (as a function of P and Λ). Therefore, the initial guess of P and Λ may be critical. We used the following algorithm to obtain the initial guess.

**Figure 18:**
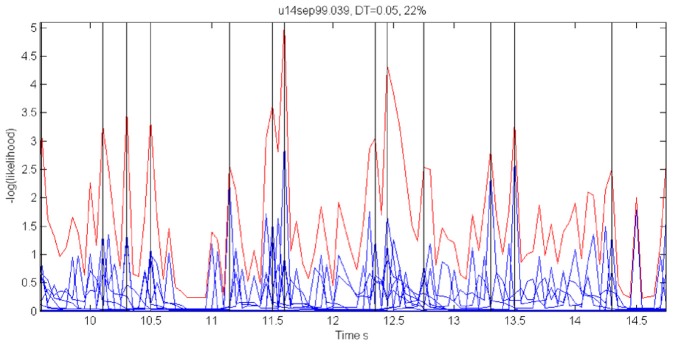
**Cooperative changes in firing rates.** Negative log-likelihood that the activity is stationary during a 5-s time interval. For all the spike trains we compute MLL_*i*_ = −log [prob(#spikes now | #spikes in the past 200 ms)] and MLL = ∑^*M*^_*i* = 1_MLL_*i*_. We show the MLL of 11 well-isolated and stable spike trains (blue traces), the sum of the blue traces (red trace) and times of transition (black lines). Top 22% of the peaks were taken as transition times. Note that several individual MLL's have peaks at the same points indicating the tendency of cooperative changes in firing rates. For each such “stationary” piece we computed the mean firing rates of every single unit obtaining a vector of M firing rates. The vectors of mean firing rates were clustered into *N* groups by the *k*-means algorithm. The probability of transition from state *i* to state *j* was estimated by counting how many times activity assigned to group i was followed by activity assigned to group *j*. The firing rate for group *i* was computed by pulling together all the time slices judged to belong to group *i*. These were, then, used to initialize *P* and Λ.

For each 50 ms window and each spike train, we computed the probability of observing so many spikes, given the number of spikes observed in the preceding 200 ms. The product of these probabilities for all the recorded spike trains provided an estimate for the likelihood that the firing rates at the present 50 ms are the same as in the previous 200 ms. Figure 18 illustrates a small stretch of such a computation.

Instead of the probability itself, we used the negative log of the probability (MLL for minus log likelihood). Peaks in MLL that exceeded five times the standard deviation of MLL were taken as points in time at which the activity flipped from one stationary state to another.

**Figure 19:**
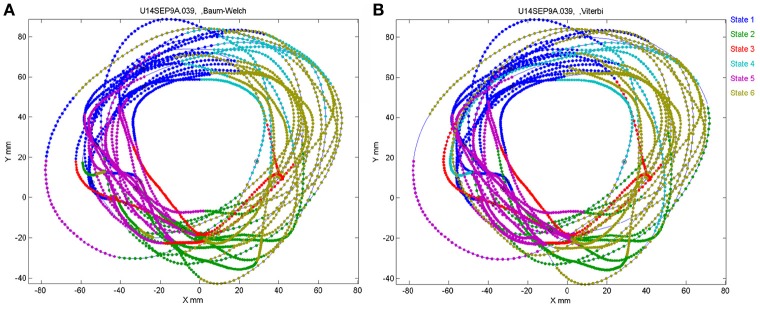
**Correspondence between drawing and HMM states.** Data for 41 s of monkey's drawing is shown. Hand position was sampled at 100 Hz. Periods defined by the HMM state are plotted in different colors. For our data Baum–Welch **(A)** provided somewhat better fit with the drawings than the Viterbi training algorithm **(B)**.

The number of states of the HMM was preselected between *N* = 6 and *N* = 8 as the results with this number of states seem to yield best results, as described below. Any series of observations will converge to some optimal P and Λ. How do we know that the HMM is a reasonable one. One way is to look at the probability of the Markov chain to be at each possible state as a function of time. A good fit to an HMM would result in sharp transients of probabilities, as may be seen in Figure 2. In the present experiments, we have the advantage of observing the behavior (scribbling) during the time that the brain activity was recorded. Thus, we may confront the series of hidden state with the drawings as illustrated in Figure 19.

In motor areas of the arm the majority of units are related to direction of movement of the arm's end-point. Indeed as seen in Figure 19 most of the time the individual HMM states map into short arches of the scribbling with similar directions.

However, we also showed in section 4 that the monkey's drawings tend to be composed of concatenations of 3 parabolas. One can hope that on some days HMM may reveal also the “intention” to draw parabolas. If unique states of ensemble activity of motor cortical cells represent movement primitives, it should be possible to associate such states with distinct movements having common characteristics. To test this possibility, we used a HMM (Abeles et al., 1995; Gat et al., 1997) and the recorded motor cortical activities were segmented in an unsupervised manner without using any information about the concurrent movements. The HMM analysis was applied to the activity of a group of simultaneously recorded motor cortical units (5–12 units/session, 8 sessions). To be considered dominant, a state had to have a probability above 0.5 for at least 0.1 s with its time-average being at least 0.75.

**Figure 20:**
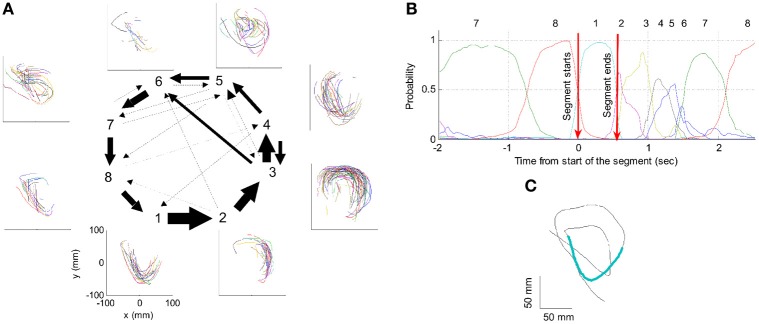
**Illustration of movement segmentation according to a Hidden Markov Model (HMM). (A)** An example of HMM results for one session. Center: Transition probabilities between states. The thickest arrows correspond to the highest probabilities, and the dashed and dotted arrows correspond to gradually lower probabilities. The largest depicted probability is about 15 times higher than the lowest depicted probability. Periphery: paths corresponding to the states (for presentation purposes, the paths with shortest and longest durations within each state were omitted so only 90% of the paths are shown). This part is reproduced in Figure 3. **(B)** Only a short time period of the data is illustrated. The HMM model, learned here for eight states, provides posterior probabilities as temporal functions. The neural activities can then be segmented based on the dominant probabilities. The numbers above the plot correspond to the state with the highest instantaneous probability. **(C)** The movement corresponding to the analysis in **(B)**. The segment based on state 1 is highlighted; this segment is parabolic.

One such segmentation is demonstrated for the results shown in Figure 20A. The HMM provided the a posteriori probabilities of the states as a function of time. Figure 20C shows a period when state 1 was dominant. Movement periods associated with the periods of dominance for the eight identified states were identified by finding an optimal time lag between the neural state and the corresponding movement segments. An optimal time lag for each state was determined by seeking a time lag providing the highest similarity between the geometrical shapes of the movement segments associated with each state lagged relative to the neural activity. A single time lag was used, although many units were active during each state and different neurons may have diverse time lags (Moran and Schwartz, 1999; Stark and Abeles, 2007). The paths identified with each state are depicted in Figure 20A. Nearly 50% of duration of the neural data analyzed in this session was identified with the periods of dominant a-posteriori probabilities of the hidden states. States 1–4 corresponded to geometric strokes easily identifiable as parabolic strokes with specific orientations. State 1 (Figures 20B,C), for example, corresponded to parabolic strokes having an orientation of 270 degrees (direction of the normal at the vertex). States 1 and 2 could be identified with single parabolic strokes, whereas states 3 and 4 corresponded to elements from sequences composed of two parabolic strokes. States 5–8 corresponded to slower movements, presumably associated with periods of rest. Thus, the HMM segmentation, although unsupervised, resulted in partitioning the movements into sets of parabola-like elements.

## 6. Time precision of neural data

In the cerebral cortex, where each nerve cell is affected by thousands of others, it is a common belief that the exact time of a spike is random up to an averaged firing rate. Precise time relations of several neurons have been observed in brain slices (Ikegaya et al., 2004) and in behaving animals. In behaving monkeys, the time intervals between spikes, measured in correspondence to a specific behavior, may be controlled to within the milliseconds range with the best case reaching 0.5 ms. The realization that time relations among different neurons could be precisely controlled and read out, can also imply that complex representations could be built from simpler ones efficiently and very fast. We used data-mining techniques and rigorous statistic testing to test how precise can time intervals between spikes of different neurons be.

### 6.1. Data mining on the spikes recording

Single-unit activity was recorded from 8 microelectrodes inserted into the motor and premotor cortices of a monkey while it was freely scribbling as described in section 3. Spike data analysis was carried out for two sets of measurements. In the first set (consisting of three experimental days), time resolution of recording was 1 ms, while in the second set (consisting of five other days) it was 0.1 ms.

The basic entity of the neural data is a single spike generated at a specific time by a specific neuron. A neural component was defined as a triple (*n*1,*n*2,δ), where *n*1 and *n*2 are two neurons and δ is a time-interval (between spikes generated by these neurons). In this way, each pair of spikes in the neural data could be interpreted as an occurrence of some neural component. For the results given in this article, the total number of time-intervals between spikes was limited to 50. In the first set of measurements, time-intervals were quantized to 2 ms. In the second set, in which spikes were recorded with a resolution of 0.1 ms, the bin width was 1 ms.

Spike sorting by shapes that were recorded through a single electrode can result in confusions. Intracellular properties which may generate precise time intervals can be confused with precise timing which is generated by the organization of activity in the network. Therefore, we considered only neural components consisting of two neurons that were recorded through different electrodes. For example, if we have three electrodes recording spikes from two neurons each, there are 30 − 6 = 24 valid pairs of neurons (note that the pairs <*n*1,*n*2> and <*n*2,*n1>* are different). Combining with the 50 possible time intervals per component, we have 24·50 = 1200 potential neural components, some of which are frequent while others may never occur. In the days analyzed there were thousands of such neural components.

### 6.2. Data mining on the drawing recording

The hand position was sampled 100 times per second (dots in the trajectory drawings). The monkey mostly drew in a counter clockwise direction. In order to find repeated patterns of drawing we used data-mining techniques. For this purpose the continuous drawing must be converted into a sequence of events (drawing events). In one experimental day there are hundreds of such events. Searching for repeating sequences of events is greatly facilitated by algorithms of data-mining. A drawing event was marked as occurring at the time at which a certain drawing-property changed from one range of values to another. The property itself may be arbitrarily chosen. For example, it could be defined as a change in the drawing direction from a range of 0-30° to a range of 30-60°. Other definitions can be based on changes in the curvature or in the velocity of the drawing. Once a set of criteria for identifying drawing events is defined, the drawing data is translated into a sequence of these events along the time axis. Then, data-mining algorithms are activated to detect repeating subsequences in the translated data. The repeating subsequences are called drawing components. Naturally, different definitions of the set of criteria lead to different drawing components.

**Figure 21:**
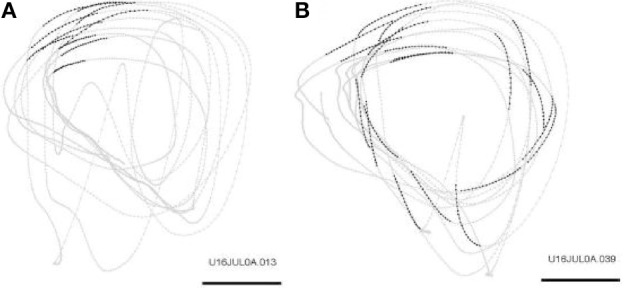
**Drawing components.** Examples of two drawing components during scribbling for 30 s. Scale bar is 50 mm. **(A)** Occurrences of the drawing component: “Transitions of drawing direction from a range of 180–210° to a range of 210–240°” are marked by larger dots. **(B)** Occurrences of the drawing component: “Transitions of drawing velocity from a range of 20–30 cm/s to a range of 10–20 cm/s” are marked by larger dots.

Repeated scribbling paths were extracted by data-mining algorithms. These paths are called drawing components. In a typical day there are 12–22 such drawing components. Figure 21 illustrates the monkey's drawings and two simple drawing components.

### 6.3. Comparison

**Figure 22:**
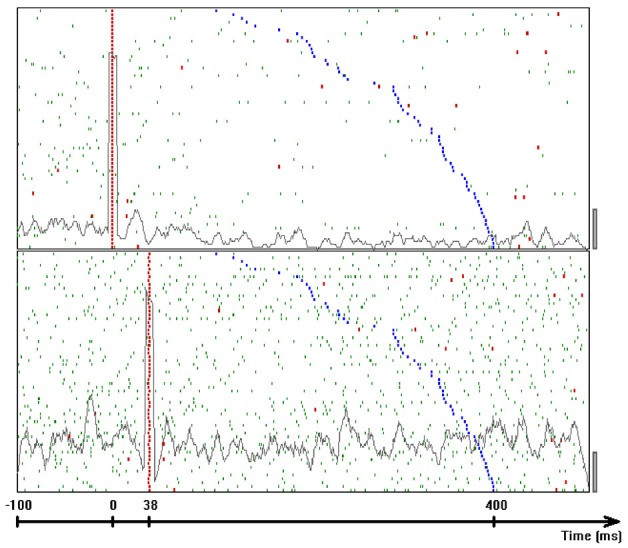
**Frequent inter-spike intervals.** Dot display showing occurrences of a frequent inter-spike interval around occurrences of the drawing component that was shown in Figure 21B. **Top panel** shows the firing times of unit 8.1. The **bottom panel** shows the firing times of unit 1.2. Each linelet represents a single spike. Linelets representing spikes which took part in the selected interval are colored red. The rasters were aligned on the first spike of the selected interval. The time of onset of the drawing is colored blue. Trials are sorted by increasing delays between the neural intervals and the drawing components. The gray line in each panel represents the average firing rate considering all 372 common occurrences using bins of 9 ms. Scale bars at the bottom right corner of each panel are 50 spikes per second.

To determine whether there are precise timing relations between the spikes of two neurons and the drawing, we selected a time slice before the start of each drawing component. For a given pair of neurons, we counted how many times a spike in the first neuron was followed by a spike in the second neuron within each of 50 particular time intervals. For the first set of measurements, these intervals were: 0–1 ms, 2–3 ms,…, 98–99 ms and for the second set they were: 0–0.9 ms, 1–1.9 ms, 2–2.9 ms,…, 49–49.9 ms. The interval that repeated the largest number of times was hypothesized to show precise firing times in relation to this particular drawing component. For example, the interval 37–38 ms between neuron 1 from electrode 8 (denoted by 8.1) and neuron 2 from electrode 1 (denoted by 1.2) repeated 372 times within the time window 400 ms to 100 ms prior to the drawing component that is shown in Figure 21B. Figure 22 depicts 62 of these repetitions (uniformly distributed such that 1 of each 6 is shown).

To assess the probability of chance events we generated 1000 surrogate spike trains by randomly teetering the time of each spike within 10 ms around its real time. For each of these surrogates we used the same idea for counting all possible intervals between neurons 8.1 and 1.2, which were repeated around the same drawing component during the same time slice. Similarly, the maximal frequency of these intervals was taken as a representative of that surrogate. For the 2 single units whose firing is illustrated in Figure 22, the 1000 maximal frequencies tended to be significantly smaller than 372. Their mean and their variance were used to estimate the probability of these 372 repetitions, assuming normal distribution of counts. For this drawing component and pair of neurons the mean was 350.7 and the standard deviation was 5.0, yielding a probability of 0.00001.

The counting process is not likely to be distributed normally, so that assessing probability by the mean and variance may be misleading. A much more complicated issue involves finding a rare event for all twelve drawing components recorded that day. We analyzed all the 50 possible pairs of neurons on that day, all 50 possible time intervals for each pair, and for seven different time slices around the start of each of the drawing components. Picking the rarest event out of all these possibilities should yield a highly unlikely event. Hence, we need to assess the likelihood of finding such low probabilities when multiple trials are conducted.

Once we had the time occurrences of all the drawing components and the neural components, we were interested in finding relations between drawing components and pairs of neurons. For each drawing component A and for each possible pair of different neurons <*n*1,*n*2> we counted the occurrences of each neural component around A that consisted of a spike from *n*1 and a spike from *n*2. In other words, we were interested in the total occurrences of each relevant time-interval between a spike of *n*1 and a spike of *n*2. The time regions in which we counted these intervals were determined relative to the occurrences of A by two external parameters *T*from and *T*to. Formally, suppose that during a recording day A occurred at {*T*1,*T*2,*T*3,…,*Tn*}, then the time regions are [*T*i + *T*_from_, *Ti* + *T*_to_], where 1 ≤ *i* ≤ *n*.

Eventually, we defined the support of a relation to be the total number of occurrences of the most frequent interval. Note that in practice the supports of the relations were computed for several [*T*_from_,*T*_to_] windows. For the first set of measurements the windows were: {[−1.4,−1.1], [−1.2,−0.9], [−1.0,−0.7],…,[−0.2,0.1]}, while for the second test, they were: {[−1.0,−0.9],[−0.9,−0.8],[−0.8,−0.7],…, [−0.1,0.0]} At a later stage, the range with the strongest result was selected for each recording day.

Figure 22 shows the spike activity around the appearances of the drawing components in Figure 21A. This figure provides further indications that the relations between the neural interval and the drawing component were not random or due to trivial artifacts: The delay between the neuronal component and the drawing component (blue marks) is not evenly distributed between −0.4 and −0.1 s as might be expected for chance relations. Second, in the lower panel the firing rate is stationary. In this condition, had the red marks been random, the spike density around these dots marks should have approximated the autocorrelation function which must be symmetric. However, the little troughs on both sides of the peak (relative refractoriness) are not symmetric. The difference is significant at 0.013.

Using statistical analysis we showed that the probability for synchronization in the scribbling—neural activity is more than 99.8%. Using the computed support for each relation in a recording day, a statistic called the *relations-score* was extracted for this day (details are given below). Intuitively, relations-score gets larger as the likelihood of the support for the relations decreases. Once a relations-score was computed for the actual data (denoted by *S*0), we evaluated its probability of its occurring by chance.

As relations between hand motion and firing rates of neurons have been studied extensively, we wanted to test whether *S*0 was significantly higher than what we would expect from random data that have similar firing rates. In order to simulate random data that preserves firing rates of neurons, we randomly teetered the time of each original spike within a small window of size W (Geman et al., 2000; Amarasingham et al., 2003).

For example if *W* = 10 ms and the original time of some spike was 125 ms, its new time after teetering may be any time within (120, 130 ms). Using this technique, we generated 5000 such surrogate spike trains (when the first set of measurements was analyzed) or 1000 such surrogates (when the second set was analyzed). Each surrogate train was given a relations-score (*S*1, *S*2, *S*3, … respectively) following exactly the same procedure as for computing *S*0 (including: re-teetering for 1000 times to evaluate the probability of each relation, re-selecting the best support among all possible intervals, and re-selecting the best time slice [*T*_from_,*T*_to_] that leads to the largest relations-score). These 5000 values were used for estimating the probability [denoted by *p*(*S*0)] of getting the value *S*0 by chance. For example, if only 50 surrogate trains exceeded the relations-score of the actual data, then *p*(*S*0) was estimated by 50/5000 = 1%. Table 1 shows the minimal width of the teetering window for each day that led to a significant value of *p*(*S*0).

**Table 1:** **Minimal widths of teetering windows**.

**First set of measurements**		**Second set of measurements**	
**Day**	**Minimal width of teetering window**	**Day**	**Minimal width of teetering window**
16 Jul 00	3 ms	26 Aug 99	0.5 ms
28 Jun 00	6 ms	14 Sep 99	3 ms
27 Jun 00	12 ms	22 Aug 99	4 ms

The table shows minimal widths of the teetering windows used in each of the six (out of eight) recording days to obtain a value of p(S0) which was less than 5%. The second set of measurements, where spike times were recorded at a resolution of 0.1 ms (instead of 1 ms), led to much better precision. Note that no significant results were achieved when smaller teetering windows were used.

A significant value for this probability indicates that the relations between drawing components and pairs of neurons (in that day) are damaged as a result of teetering within a window of size W. By this we can conclude that around the occurrences of similar behavior, pairs of spikes tend to prefer specific Inter-Spike Intervals (ISIs). We considered only which ones were involved in significant relations (i.e., relations with an estimated probability less than 0.05). Note that due to the fact that only a minute set of neurons in the cortex is recorded, not necessarily all the recorded neurons would show the real time accuracy of the brain.

A solution which can be used to test the null hypothesis that spike times are random within a window of width W was offered (Geman et al., 2000; Amarasingham et al., 2003). If this null hypothesis is true, then replacing the time of each spike by a randomly selected time within W around its true time should not affect any of the statistics extracted from the spike times. To use this idea we need to describe the entire set of relations between firing intervals and drawing by one statistic. To do so we defined a statistic based on the ten least likely relations between pairs of spikes and any of the drawing components. We termed this the *relations-score*. Intuitively, relations-score gets larger as the existing relations are less likely to exist by chance. For each recording day we computed a relations-score for the actual data. Then we randomly teetered all spike times within some time window W and recomputed it for the teetered data. Given a teetering width W, the computation of the relations-score statistic for a recording day involves the following steps:

1. Generate a set of 1000 independent teetered neural data (denoted by *J*_1000_) by teetering the actual data within the window W. Observe that the same W was used to create both *J*_1000_ and all surrogates for which we re-calculated the relations-score.
2. Recognize all potential relations between a drawing component and a pair of neurons.
3. For each relation R do the following:
  - Compute the support of R (denoted by *R*_support_).
  - Skip R if its support is less than a predefined noise threshold. This step is carried out in order to prune noisy relations. Note that the values used for this threshold were 60, 40, 20, 20, 30, and 30 for the six significant recording days listed in Table 1 from left to right respectively (depending on the firing rates of the neurons in that day).
  - Compute the support of R for each neural data in *J*_1000_ (using the same drawing component).
  - Compute the mean μ and the variance σ^2^ of these 1000 values.
  - Estimate the probability of *R*_support_ assuming the normal distribution *N*(μ, σ^2^).
  - Set *R*_surprise_ to −log_2_ *P*(*R*_support_).
4. Sort all relations by descending order of their computed *R*_surprise_. If several relations involve the same two neurons and the same interval between them, delete them all except from the first (the one whose *R*_surprise_ is the highest).
5. Set relations-score by the sum of the *R*_surprise_ values of the first 10 relations.

**Figure 23:**
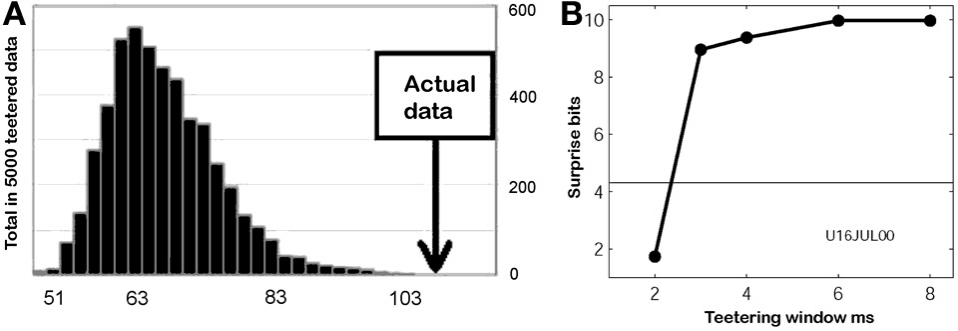
**Relations-score and teetered data. (A)** Distribution of relations-scores for surrogate spike trains and the actual data. Five thousand surrogate spike trains were independently generated by teetering spike times within 10 ms. For each of these a relation-score was extracted. The distribution of these relations-score values was estimated by a histogram. The actual data had a value of 106.37 (arrow). None of the 5000 surrogate trains had a value above it. Hence the *p*-value for the actual data was estimated as less than 1/5000. **(B)** Surprise values for different teetering windows. Abscissa is the teetering window, ordinate is the surprise value. The horizontal line shows the surprise value for significance of 0.05. Thus, teetering within 3 ms already had a significant effect.

Independent teetering was done 5000 times (for the first set of measurements) or 1000 times (for the second set), and a histogram of the relation-scores for teetered data was constructed. After each teetering all the parameters for extracting the relations score were re-evaluated to obtain the highest possible value for each teetered data set. Figure 23-left illustrates this histogram for *W* = 10 ms. As stated above, all computations of relations score for each teetered data set were performed de novo by the same process; the same was done for the actual data (including multiple trials of all possible drawing components, pairs of neurons, time intervals and time slices).

This method was used to estimate the probability *p* of the relations-score value of the actual data. From this probability we derived the surprise-value defined as −log_2_ (*p*). Figure 23-right shows the surprise values for one recording session, obtained when spike times were teetered within different windows between 2 and 8 ms. Clearly, teetering within 3 ms already had a significant effect on the surprise value. Thus, Figure 23 right already indicates that the spike times of the cortical neurons are accurate within 3 ms. Although we used 2 ms bins for measuring the intervals in that day, note that teetering by 2 ms is not pointless, due to the fact that intervals are binned only after teetering is done. Since the original data are measured at a resolution of 1 ms in this case, intervals may be still damaged by teetering.

Significant relations-score values were observed in 3 days (out of three) for the first set of measurements and in three (out of five) for the second set in this study (total of six significant days). For the 3 days of the first set, the smallest teetering windows producing significant results were 3 ms (shown here), 6 ms, and 12 ms. For the 3 days of the second set these windows were 0.5, 3, and 4 ms. Note that this represents an upper bound for the resolution. One may find that a different statistic can indicate even higher time precision.

When the same procedure was repeated step by step for the neural data around randomly selected points in time (instead of time occurrences of drawing components), no significant surprise values were found (for a teetering window W of 10 ms). Thus, we only obtained significant time relations by relating the neural intervals to specific features in the behavior. Furthermore, no significant surprise values were found when the same procedure was repeated taking a teetered neural data instead of the original data.

In section 7 we will present a specific implementation of a neural architecture, the synfire chain (Abeles, 1991) that provides an explanation for the observation of these timing relations.

## 7. Modeling neural movement control with synfire chains

### 7.1. Synfire chains

Here we describe neural network simulations of synfire chains and consider in particular how multiple synfire chains may be embedded in a large network. Synfire chains can be seen as constituting sub-networks of the local cortical network and interact in the context of the ongoing background activity. The original version was a chain of length *l* composed of groups of *w* neurons each (the chain width), with full unidirectional connectivity between successive groups. A group together with its output connections can interchangeably be called a link in the synfire chain. Such a chain structure propagates near-synchronous activity in each group along successive groups like a row of dominoes. Detailed properties of various versions of such systems have received considerable theoretical attention; stability properties (Herrmann et al., 1995) have been studied as well as structural variations such as partial group to group connectivity or such as feedback when a given neuron occurs in more than one group. There also have been studies of partially interconnected synfire chains to define the conditions in which activity can jump between chains (Hayon et al., 2005).

**Figure 24:**
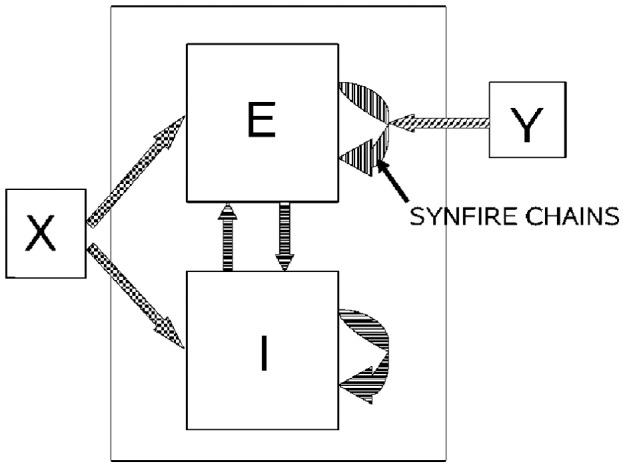
**Sketch of the cortical network model.** All connections between the 40,000 excitatory neurons in the model network are formed by synfire chains (vertically striped arrow). A neuron can occupy only one position in each chain but may contribute to several ones out of the total of 50 chains. Each chain consists of 20 pools each of which is fully connected to the next pool. The intra-chain synapses enable excitatory postsynaptic potentials (EPSPs) of *a* = 0.5 mV generated by alpha-function current with a rise time τα = 0.2. Synaptic delays are drawn from a uniform distribution between 0.5 and 3 ms, but are identical for all synapses connecting one pair of pools. Chains are stimulated at 1 Hz with independent Poisson sources. Stimuli arrive only at the neurons in the first pool of a chain and consist of 100 spike times drawn from a Gaussian distribution with standard deviation σ = 1 ms. In addition to the excitatory (E) neurons there are 10,000 inhibitory (I) neurons which are recurrently interconnected (horizontally striped arrows): each neuron establishes a random number of synapses drawn from a binomial distribution with the mean given by 10% of the size of the respective target population. EPSP amplitudes outside chains are *a* = 0.1 mV, inhibitory postsynaptic potential [IPSP] amplitude are 6-fold larger and have a rise time of 0.6 ms. Delay distributions are the same as within chains. Each neuron receives (cross-hatched arrows) an excitatory DC drive of 350 pA. Further parameters for the integrate-and-fire point neurons are τ = 20 ms, *C* = 250 pF, θ = 20 mV, τref = 2 ms. Excitatory neurons are randomly chosen to form 50 completely connected synfire chains (vertically striped arrow). A neuron can occupy only one position in each chain but may contribute to several chains. Intra-chain synapses are 5-fold stronger than other EPSPs. Intra-chain delays are drawn from the same distribution as others but are identical for synapses connecting a particular pair of neuron links. Chains are stimulated at 1 Hz with independent Poisson sources. A stimulus consists of 100 spike times drawn from a Gaussian distribution with standard deviation σ = 1 ms and is received by all the neurons of the first link in a chain.

For a theoretical framework, we will refer to the balanced random network architecture which is a common single layered model of the local cortical network (van Vreeswijk and Sompolinsky, 1996; Amit and Brunel, 1997; Brunel, 2000). It explains the asynchronous irregular low rate activity of cortical neurons and the large fluctuations of the membrane potential observed *in vivo* and will serve as the basic architecture also here, see Figure 24. Our network (Figure 24) differs from previous approaches in two essential properties: First, there is heterogeneity in the number of dendritic synapses. Second, the excitatory-to-excitatory sub-network is purely composed of synfire chains.

The dynamics of the network is adjusted to the asynchronous irregular (AI) regime to reproduce relevant aspects of cortical dynamics: Low-rate firing of the individual neurons with Poisson-like interval statistics and large fluctuations of the membrane potential. The model consists of 80% excitatory and 20% inhibitory integrate-and-fire point model neurons (see Figure 24 for the full set of parameters). For the E-I, I-I, and I-E connections the neurons establish a random number (drawn from a binomial distribution) of dendritic synapses. The mean of the distribution is a tenth of the size of the possible connections which does not exclude multiple synapses between a pair of neurons. The excitatory-to-excitatory sub-network is here, in contrast to other works, purely composed of a superposition of *m* = 50 synfire chains. These chains are consecutively constructed. First, we randomly select *wxl* neurons (here: *w* = 100, *l* = 20) from the population of excitatory neurons without repetitions. In the next step, these neurons are connected into a feed-forward sub-network of *l* successive links of *w* neurons. Each neuron, except the neurons in the first link, establishes a dendritic synapse with all the neurons in the preceding group. The connection procedure is repeated *m* times and leaves us with complete divergent and convergent connectivity between the links (Griffith and Horn, 1963; Abeles, 1982).

An excitatory neuron may participate in several chains but occurs at most once in any given chain. The synaptic delays are drawn from the distribution specified earlier for the other synapses but is kept fixed for all synapses connecting two subsequent links of neurons. The amplitudes of postsynaptic potentials of synapses in the chains are fivefold larger than those of other excitatory connections. In the absence of specific stimuli the synfire chains are not active and the excitatory neurons spike irregularly at a low rate. For the data presented herein we apply independent 1-Hz Poisson trains of stimuli to the synfire chains. A stimulus consists of a volley of 100 spikes. The individual spike times are drawn from a Gaussian distribution centered at the time of stimulation with standard deviation of 1 ms. This volley is sent to all the neurons of the first group in a chain using synapses as strong as the intra-chain synapses.

Synfire activity stably propagates, but several chains can be active simultaneously. With a load of 50 chains the dynamics is still stable, although the global activity exhibits large fluctuations mainly due to the initiation and termination of chains.

**Figure 25:**
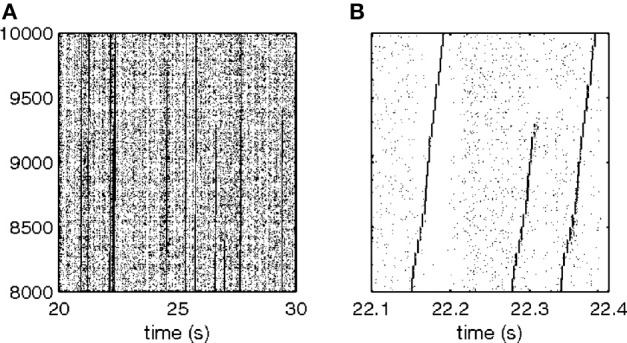
**Raster representation of the activity of the neurons in a particular synfire chain. (A)** The randomly assigned neuron numbers have been remapped so that the 2000 neurons of the chain are labeled 8001–10,000 (ordinate). The data show an arbitrary 10-s segment (abscissa) of activity. The thin black, almost vertical lines represent runs of this chain. **(B)** Temporal magnification of a portion of **(A)**. Two complete runs and one partial run of the chain are shown. At this timescale the nearly synchronous activity of each link in the chain becomes visible, as does the propagation time between links.

In the neural net simulations we deliberately randomized the identity of neurons with respect to the embedded synfire structures to mimic the random sampling in real recordings. It is instructive, however, to remap some data so that adjacent lines in the raster are associated with particular links and chains. The strong, nearly vertical lines in the raster are firings of the synfire chain, background neuron firings in the raster represent either spontaneous activity or the activity of other chains, since each neuron in this chain is also a member of two to three other chains. Figure 25B shows a portion of this raster with high time resolution revealing details of several chain runs. The offset between short vertical segments corresponds to activations of successive links; the offset is the interlink propagation or synaptic delay. Note that failure of propagation is possible such as shown in the middle run of the chain.

**Figure 26:**
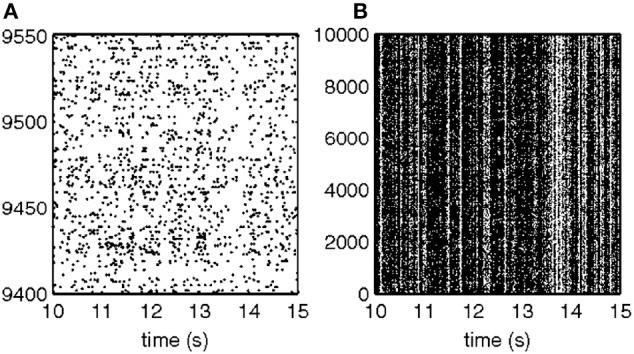
**Fluctuations in network activity. (A)** Raster of 150 randomly selected neurons (ordinate) during a period of 5 s (abscissa). The activity of the neurons appears uncorrelated, irregular, and fairly stationary in time. **(B)** A raster of 10,000 neurons during the same time period as in **(A)**. The activity exhibits prominent vertical bands.

Without the remapping of neuronal identities (Figure 25) synfire chain sequences are not visible in raster-plots. Figure 26A shows a 5-s segment of the activity of 150 excitatory neurons randomly selected (and not remapped according to chain membership) from a synfire simulation (50,000 neurons, 50 fully connected synfire chains independently stimulated by low-frequency Poisson trains); the raster has no particular structure. The raster from a larger set of 10,000 excitatory neurons over the same time range is shown in Figure 26B. Again, since the neurons are chosen randomly, there are no structures like those in Figure 25B that are directly ascribable to activations of individual synfire chains. However, there is an obvious broader vertical striping that connotes a coordinated fluctuation in the population activity; this striping becomes increasingly visible when the raster display shows a large number of neurons (contrast Figure 26A vs. B). It turns out that these coordinated rate fluctuations are the result of fluctuations in the number of synfire chains that have been activated at any time. Individual neurons, however, experience no rate increase.

**Figure 27:**
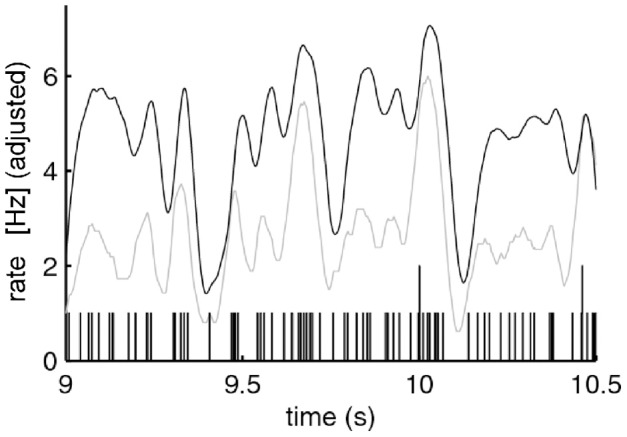
**Relation of population firing rate and synfire activation rate.** The black curve represents the smoothed population activity (triangular filter, full width 51 ms); the gray curve represents smoothed stimulation times (same filter) of all synfire chains. The actual stimulation times are shown as vertical bars at the bottom.

Figure 27 juxtaposes (1) the times of chain stimulations with (2) their (low-pass) temporal representation (i.e., the number of chains activated at any time) and (3) the fluctuations of total population rate. These are clearly strongly, although not completely, related.

The framework described so far combines general ideas about the dynamics of neural activity in the cortex and observations of precise firing patterns related to motor activity. We will now formulate a model that supports the evidence for such relations. An obvious question is now how movement primitives are represented in a dynamic network of spiking neurons.

It has previously been shown that networks of synfire chains can generate sequences of primitives in an abstract model (Schrader et al., 2010; Hanuschkin et al., 2011). We will consider a functional model that is simultaneously capable of reproducing several experimental findings on cortical activity and generating trajectories which exhibit key features of free monkey scribbling. Recent theoretical studies suggest that coupled synfire chain structures can demonstrate compositionality, the hierarchical representation of complex entities in terms of parts and their relations (Abeles, 1982; Hayon et al., 2005).

### 7.2. Movement representation model

The model consists of a topologically organized network of synfire chains. Neurons in the same pool of a chain encode the same preferred velocity vector, thus realizing a population coding for movement (Georgopoulos et al., 1986). The trajectories generated by our model consist of a series of parabolic segments similar to those identified experimentally (Polyakov et al., 2001, 2009b) which fulfill the well-established two-thirds power law relationship of velocity and curvature (Lacquaniti et al., 1983; Viviani and Flash, 1995). It has previously been demonstrated that monkey scribbling is well approximated by parabolic strokes (Polyakov et al., 2001, 2009b). Parabolic movement primitives obey the two-thirds power law, are invariant under equi-affine transformations and minimize end effector jerk.

A parabola can be constructed from a constant acceleration produced by a homogeneous force field. Assume the initial position and velocity of a point mass is *X*^0^ = (*x*_0_, *y*_0_)^*T*^ and *V*_0_ = *Ẋ*_0_ = (*ẋ*_0_, *ẏ*_0_)^*T*^, respectively. If the point mass experiences a constant acceleration *a* = (*a*_*x*_, *a*_*y*_)^*T*^ then the trajectory of *x* = (*x, y*)^*T*^ is given by

$$x(t)=x_{0}+v_{0}t+\frac{1}{2}at^{2}$$

The curvature of the path is

(4) $$c=\frac{\left|\dot{x}\ddot{y}-\dot{y}\ddot{x}\right|}{v^{3}}$$

where *v* = ∥*ẋ*∥ is the tangential velocity. Since |*ẋÿ* − *ẏẍ*| = |*ẋa*_*y*_ − *ẏa*_*x*_| is constant, c and v obey the following relation

(5) $$v=Kc^{\frac{1}{3}}$$

where *K* is named velocity gain factor. Equation 5 is thus satisfied by parabolic movement segments.

Constant accelerations are equivalent to velocities that change linearly in time, as $a=\frac{dv}{dt}$. In velocity space, linearly evolving velocities ($\dot{v}=\text{const}$) are represented by a uniform motion along a straight line. Therefore, straight lines in velocity space can be mapped into parabolic trajectories in position space.

A neural architecture which can be associated with a uniform motion is the synfire chain (SFC) (Abeles, 1991). In the simplest formulation of the SFC concept, excitatory neurons are grouped in pools and each neuron is connected to all neurons of the following pool creating a chain of convergent and divergent feed-forward connections. If the first group is stimulated with sufficient strength and a sufficiently high degree of synchrony, a wave of synchronous activity propagates along the chain. The propagation along the chain is at constant speed (Wennekers and Palm, 1996; Diesmann et al., 1999) and stable under fairly general conditions (Herrmann et al., 1995).

An appropriate mapping of preferred velocities to the pools of a synfire chain enables the generation of an individual parabolic segment. By extension, a series of parabolic strokes in position space can be realized by uniform motion along a graph of connected straight lines in velocity space. Each arrow in velocity space is realized by a synfire chain with the corresponding velocity mapping. By construction, the velocity at the end of one parabolic stroke is equal to the velocity at the beginning of the next. When the activity volley in a synfire chain reaches the final pool, feed-forward connections to the initial pools of the two potential successor chains initiates the propagation of an activity volley in each of them. Assuming a strong competition between the two stimulated chains, such that only one of the chains can continue propagating the activity, a trajectory of parabolic segments is produced. We analyze the properties of the generated trajectories to see whether they are sequences of parabolic segments in accordance to the above remarks on equi-affine geometry.

The activity of single cells in the motor cortex has been shown to be directionally tuned to arm movements (Georgopoulos et al., 1982). The arm trajectory can be estimated by calculating the population average over all neurons (Georgopoulos et al., 1986, 1988). Similarly, we use population coding to generate a trajectory from simulated neuronal activity:

(6) $$v=\sum_{k}^{\text{neurons}}w_{k}a_{k}(t)p_{k}=\sum_{i}^{\text{chain}}\sum_{j}^{\text{pool}}w_{i}^{j}a_{i}^{j}(t)p_{i}^{j}$$

where ***v*** is the instantaneous velocity, *a*^*j*^_*i*_(*t*) is the activity in the *j*^*th*^ group of the *i*^*th*^ chain and *p*^*j*^_*i*_ its i preferred velocity. The weights are set to *w*^*j*^_*i*_ = 0.02s ∀*i, j* resulting in velocities comparable to the monkey experiments [median 300 mm/s as given by Polyakov et al. (2009a,b)]. The propagation speed of the activity volley in an SFC from one pool to the next is constant as described above. We can therefore map an SFC to an arrow in the velocity space. Each pool of the SFC is assigned its preferred velocity *p*_*j*_ according to its position along the arrow, i.e., for a chain consisting of *n* pools mapped to an arrow starting at *v*_0_ and ending at *v*_1_,

(7) $$p_{i}=\frac{i-1}{n-1}\left(v_{1}-v_{0}\right)+v_{0}$$

**Figure 28:**
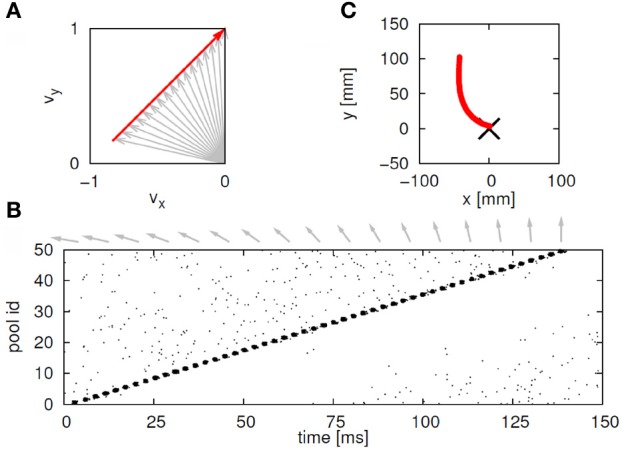
**Mapping synfire activity to parabolic movements. (A)** The preferred velocity vectors for the pools of the synfire chain (gray arrows; shown for every third pool of the chain) are determined by sampling a straight line in velocity space (red arrow). **(B)** The spiking activity of an activity volley propagating with constant speed along a synfire chain. Preferred velocity vectors for every third pool as in **(A)** are shown as gray arrows above the dot display. **(C)** Generated parabolic trajectory. The black cross at (0,0) indicates the start position.

This is illustrated in Figure 28A; the activity of the corresponding synfire chain is given in Figure 28B. As the preferred velocity for each chain *j* changes linearly with the pool index *i* and the propagation speed from one pool to the next is constant, the instantaneous velocity vector also evolves linearly resulting in parabolic motion as derived in section 7.2. Figure 28C shows the parabolic trajectory in position space generated by the synfire activity in Figure 28B. To extract the trajectory from the simulated neuronal activity, we bin the activity in 1 ms intervals and reconstruct the motion according to the population coding scheme given by Equations 6 and 7.

### 7.3. Competition and cooperation between chains

We develop a spiking network model to realize a generator of random trajectories consisting of parabolic segments. Our model comprises two interconnected networks. The synfire chain network (SFCs) consists of ten chains, each chain corresponding to one of the arrows in velocity space and thus encoding a parabolic segment. Each chain consists of 80% excitatory neurons that make feed-forward connections with dilution factor *p*_d_ = 0.75 and 20% inhibitory neurons making *k*_g_ random connections to other neurons in the SFCs network. To distinguish the random inhibitory connections from other connectivity patterns, we will refer to *k*_g_ as the global inhibition parameter.

**Figure 29:**
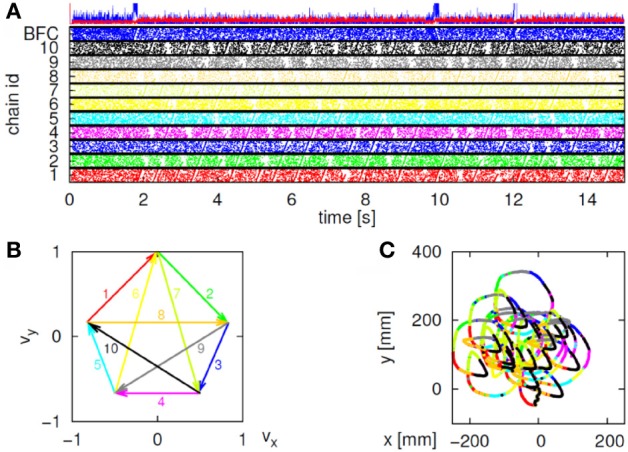
**Generation of scribbling trajectories. (A)** Spiking activity of bidirectional and synfire chain networks. Colors of the activity of each chain corresponds to the colors of the arrow in velocity space shown in **(B)**. Above the raster plot the average firing rate of the synfire network (red) and the bidirectional network (blue) is plotted. **(B)** Abstract generator for trajectories consisting of parabolic segments. Uniform motion along straight lines in velocity space is equivalent to parabolic motion in position space. Each colored arrow represents a parabolic segment and its direction of execution. When the end of an arrow is reached, one of the two successor arrows is selected. **(C)** Scribbling trajectory extracted from the spiking activity using population coding. Segments are drawn in the color of the most active synfire chain.

Feed-forward connections join the final group of each chain to the initial groups of two other chains, e.g., the final group of chain 1 has feed-forward connections to the initial groups of chains 2 and 7, see Figure 29. The preferred velocity of the last group of a chain is the same as the first groups of the chains it connects to in order to generate trajectories that are smooth at the transition points. Reliable switching at the transition points is enabled by mutual inhibition between potential successor chains.

We create a synfire chain with feed-backward as well as feed-forward connections, both with a dilution factor of *p*_d_ (a backward-and-forward connected chain, BFC). One end of the BFC makes excitatory feed-forward connections with dilution factor *p*_d_ to the initial pool of chain 1 in the SFC network. Each inhibitory neuron in the SFCs network makes *k*_*B*_ connections to neurons randomly selected from the BFC network, thus inhibiting its activity when synfire activity is present. If synfire activity is extinguished, the drop in inhibition causes a self-ignition in the unstable BFC, which in turn triggers a fresh wave of activity in chain 1. Thus, the recurrent connections between the SFCs network and the BFC network ensure sustained activity. The dynamics of the BFC network and of the interaction with the SFCs network are investigated in section 7.4. The scaling of inhibitory synapses with respect to excitatory synapses and the rate of the external excitatory Poisson input to each neuron in the SFC network are chosen such that in the absence of synfire activity, the network spikes in the asynchronous irregular (AI) regime (Brunel, 2000).

Each of the chains in the SFC network represents a parabolic movement primitive. To produce a series of primitives, it is necessary that activity reliably propagates from one SFC to exactly one of multiple (here two) potential successor SFCs at the vertices of the network graph. In our model, cross-inhibition between two competing SFCs realizes this switching between two simultaneously activated SFCs. There are two possible approaches to achieve reliable switching: (1) cross-inhibition can be structured such that synchronous activity in each pool directly inhibits the activity in the next pool of the competitor chain. This approach is motivated by the idea of synfire binding (Hayon et al., 2005; Schrader et al., 2010), in which two simultaneously active chains can bind a third chain due to structured excitation. In an alternative approach (2) the cross-inhibition is unstructured. Synfire chain competition relying solely on global inhibition has recently been proposed by Chang and Jin (2009). However, in their study the synfire chain activity is “driven”: a supra-threshold driving input is combined with dominant global inhibition. In contrast, our model exhibits activity in the asynchronous irregular regime due to balanced global inhibition (van Vreeswijk and Sompolinsky, 1996) and only exhibits synfire activity if the initial pool of a chain receives additional stimulation. Due to our different activity regime, additional assumptions on the inhibition between chains need to be made to realize reliable switching.

Each neuron in the initial pools of the potential successor chains is activated by *p*_*CE*_ randomly chosen excitatory neurons from the final pool of the preceding SFC. The symmetric connections ensure that the successor chains are stimulated equally. All inhibitory neurons of pool *i* of one potential successor chain project to *kc* neurons of pool *i* + 1 of the other potential successor chain, and vice versa. Thus, each wave of synchronous activity directly inhibits the propagation of the activity to the next pool in the competitor chain, leading to a competition. The activity in the losing chain dies away leaving the activity in the winning chain to continue propagating, thus realizing a switching mechanism.

### 7.4. Activity state transition in the BFC

When the backward-and-forward connected chain (BFC network) described above is not being inhibited by the presence of synfire chain activity in the SFCs network, the external drive is just strong enough to induce spontaneous synfire activity and so re-ignite activity in the SFCs network. The ignition of synfire activity in the network can be understood intuitively as follows: random synchronous activity in a small subset of the neurons in a given pool *i* will be projected to the pools *i* ± 1, which in turn project back to *i*, thus building a recurrent positive feedback loop. Synfire activity emerges spontaneously at around 380 ms and propagates in both directions along the chain. Once the spike volleys have reached the ends of the BFC the synfire activity is extinguished. A reflection of activity does not occur because for any activated pool, the neurons in the previously activated pool have been reset by the propagating volley. When the SFCs network has been re-ignited, the increased inhibition decreases the net drive to the network such that no spontaneous synfire activity occurs.

Synfire volleys caused by spontaneous self-ignition tend to be of limited duration. Activity volleys traveling in different directions cancel each other when they meet and volleys reaching either end of the BFC are not reflected. Furthermore, the high activity in the BFC during synfire activity results in strong global inhibition and a reset of nearly all neurons, which in turn decreases the probability of self-ignition until activity has built up again. However, if the pool size is chosen sufficiently large, a single self-ignition results in ongoing pathological high firing rates.

The spiking activity of the complete network underlying the average firing rates is given in Figure 29A. The trajectory extracted from the synfire activity is shown in Figure 29C. The trajectory consists of a long random sequence of parabolic movement primitives. Small overlaps can be seen at the transition points where both successor chains are active before one of the chains wins the competition. The distribution of the length *n* of an uninterrupted sequence is well fitted by *P*(*n*) = *p*_0_(1 − *p*_0_)^*n*^, where *p*_0_ is the probability that neither successor chain is activated during synfire chain switching.

We analyze the characteristics of the trajectory shown in Figure 29C. Due to the piece-wise constant accelerations we conclude that the trajectory does indeed fulfill the two-thirds power law (e.g., Viviani and Flash, 1995). The equi-affine curvature of the trajectory is close to 0. We therefore conclude that the trajectory does indeed consist of a series of parabolic segments.

## 8. Compositional nature of the drawings

### 8.1. Temporal properties of strokelets

We will now describe another approach to analyze the compositional nature of the drawings. Using machine learning techniques, we show how very elementary strokelets can be treated as allophones of spoken language, which can be in turn clustered into equivalence groups (like phonemes of language) and how by Hidden Markov modeling combined with the information-bottleneck technique, these groups can be composed into words. Furthermore, the syntax of sequencing these words into sentences is revealed in this analysis.

Human subjects were tested on the hidden target task as described in the methods (Section 3). Each subject was first holding the manipulandum while searching for an invisible target. Each target hit added a little sum to the subject's fee, so subjects were highly motivated to hit as many as possible targets. After a few sessions of this type, subjects were told that every time they hear a beep they should stop as fast as possible.

We postulated that if the searching motion is composed of primitives (Sosnik et al., 2007), then it would be hard to stop anywhere. Rather if the “command” to draw a primitive was already issued it will execute to completion. Indeed, it was found that typically motion continued with several peaks in the tangential velocity. Figure 10B illustrates that. The delay from the instruction till stopping was longer then delays reported for stopping a simple motion. Furthermore, in subjects where the motion-style showed repetitive patterns the piece of drawing which continued after the “stop instruction” was similar to the typically repeated pattern.

Figure 10 also illustrates the well-known phenomenon of isochrony. The duration of each hill in the drawing was approximately identical and those after the stop instruction had the same duration as those while freely scribbling. We argue that the neural networks responsible for such behavior should have similar properties: constant duration of activity and hard to stop in the middle. Indeed, the synfire chains as proposed in section 7 have these properties.

**Figure 30:**
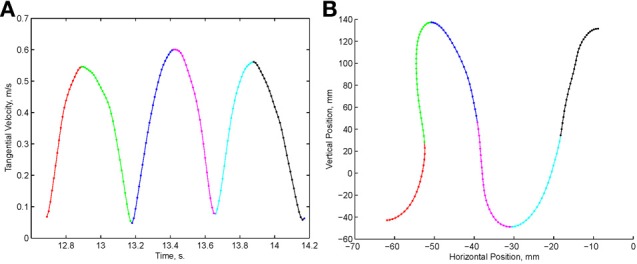
**Breaking a drawing into strokelets.** One second of human drawing is shown. **(A)** The tangential velocity. **(B)** The drawing. Each strokelet is colored differently to facilitate visualization.

To get a better glimpse at what are the elements of these drawings we broke the drawing to small pieces and studied the rules of combining the pieces into a whole. Breaking into pieces was done by considering the drawings between successive extrema of tangential velocity as drawing elements. It may look artificial to break a motion into two elements at the peak velocity. However, drawing parameters may change abruptly at points of maximal tangential velocity. Our analysis in the previous parts pointed at parabolas as being drawing primitives in monkey scribbling. These primitives too, start and end at maxima of tangential velocity. Figure 30 illustrates a little piece of the drawing and how it was decomposed into little strokelets.

### 8.2. Parsing the motion

The scribbling in one block of motion for one subject is illustrated in Figure 11. If we look on a short stretch from this block (Figure 30B), it is clear that he moves at a high speed when the trajectory is close to straight and slows down when it is curved. Figure 30A shows the tangential velocity (speed) of drawing for this section. It is composed of 3 hills of almost equal duration (isochrony).

We used this features to parse the drawing into small strokelets. Figure 30A shows the tangential velocity during 1 s of scribbling. Each strokelet spans the time between adjacent extrema and is colored differently. Figure 30B shows the actual drawing shape of each of these strokelets.

We selected 10 equally spaced points on each strokelet and computed the angle of the tangent at this point. These, ten dimensional vectors were then grouped by a Gaussian Mixture Model (GMM). By construction, an accelerating strokelet must be followed by a decelerating one and vice versa, except at the very beginning and end of the drawing. Therefore, we grouped separately the two kinds of strokelets. GMM was able to divide the strokelets into 30–50 groups.

### 8.3. Information bottleneck method

**Figure 31:**
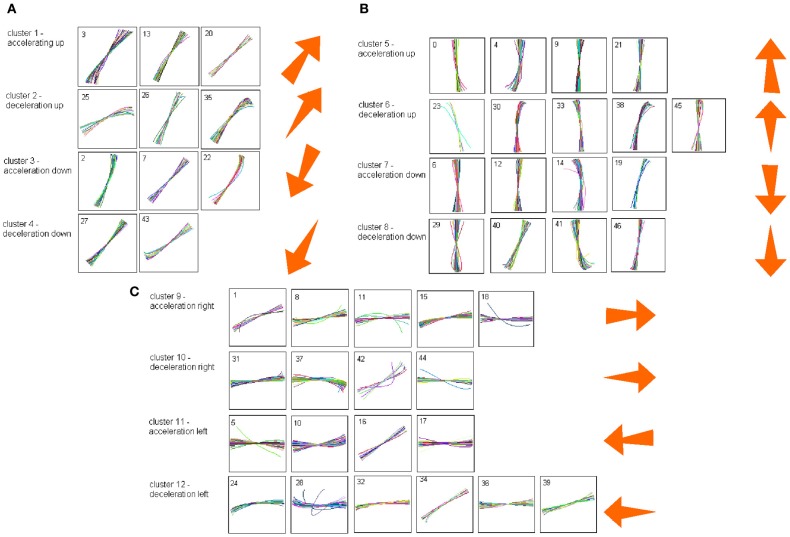
**Clustering of strokelets.** Each little panel shows all the strokelets that belong to one group. They are colored differently to facilitate visual discrimination among them. All the strokelets within the same group are stretched to the same length and plotted on top of each other. Each line of groups belongs to the same cluster. The arrow to the right of each row represents the direction of motion in this cluster. Expanding arrows symbolize accelerating strokelets and shrinking arrow represent decelerating ones. **(A)** oblique movements. **(B)** Vertical movements. **(C)** horizontal movements.

We further clustered the groups using the information bottleneck idea (Tishby et al., 1999). Suppose we deliver a large number of stimuli (X) and make observations of the responses (Y). We can ask whether we can represent X by a small number of clusters (P) such that the mutual information I (P; Y) between P and Y will be close to that between X and Y. If so, P will be the “stimulus features” represented in the responses. Alternatively, we may ask whether we can cluster Y into a small number of clusters Q, such that I (X; Q) is close to I (X, Y). If so, then Q represents a feature representation in the responses. In our case, we treat the strokelets as “stimulus” and the following strokelets as “responses.” Thus, the sets X and Y are the same. Therefore, we expect P = Q [see Erez et al. (2009) for more details]. Using this idea, we clustered the groups of strokelets into twelve clusters, see Figure 31. We can take the matrix of probabilities of transitions between strokelet-groups and reshuffle rows and columns so that all strokelets in the same cluster are at neighboring lines (and columns) in Figure 12.

Several features jump to the eye when examining Figure 12. Nonzero transitions are concentrated in patches. We observe that there are three distinct cycles. Clusters {1, 2, 3, 4} follow each other in cyclical manner and so do {5, 6, 7, 8} and {9, 10, 11, 12}. Finally transitions from one cycle to another happen only at particular groups. If we compare the drawings to language, we can say that each individual strokelet is like a phone, the 47 groups of strokelets are phonemes, the cycles are words and the sequences of words are sentences. Let us call the cycles W_1_, W_2_, W_3_, then the sentences look like: “W_1_ W_1_ W_1_ … W_1_” followed by “W_3_ W_3_ W_3_ … W_3_” followed by “W_2_ W_2_ W_2_ … W_2_.” The transition between sentences happens with specific allophones (for example W_3_ changes to W_1_ mostly by one allophone of cluster 9 to one allophone of cluster 2).

This structure can be seen readily in the actual drawings (Figure 11) where the subject is scanning the workspace repeatedly from left to right and back, then vertically and then obliquely. We note that this form of scribbling is not just less elegant than the monkey's scribbling (Figure 1), but also involves much higher accelerating—decelerating motion and larger distances between hitting targets and obtaining the reward. We conclude by stating that we found strong evidence for primitives in human scribbling and for hierarchical organization of the drawing elements.

## 9. Summary

In sections 3–8 we provided further evidence to support our working hypothesis. According to this hypothesis when a subject repeats again and again he same scribbling task, the entire scribbling may be regarded as a composition of a few elementary shapes. These shapes may be produced by activity propagating between neuron-groups. In each group the neurons have similar velocity tuning and the propagation is with approximately constant delay from group to group. When the drawing elements are parabolas they are better described by equi-affine parameters and the sequence of neuronal groups are laid along a straight line in velocity space. We provide some evidence that for neurons that are tuned to the speed of motion equi-affine speed is somewhat more adequate descriptor then the Euclidian speed.

The synfire chain model is a simple network that may have the required properties. We show that it is feasible to generate drawings similar to that of a monkey by such networks. We also provide indirect support to this idea by showing that one can detect precise timing relation between pairs of neurons in relations to the drawings.

Finally we show how the preferred sequence of such elements in the drawings can be revealed, and suggest that such preferred sequences are generated by partial connections among synfire chains.

The supporting evidence given here is partial and there is room for considerable more experimental and theoretical work on all the points suggested here.

### Conflict of interest statement

The authors declare that the research was conducted in the absence of any commercial or financial relationships that could be construed as a potential conflict of interest.
